# Nanocellulose Alleviates Intrahepatic Cholestasis of Pregnancy via Gut Microbiota‐Mediated Bile Acid Homeostasis

**DOI:** 10.1002/advs.202518337

**Published:** 2026-06-09

**Authors:** Muhua Yu, Hongjie Dai, Xiaocui Zhong, Qibin Li, Hui Yuan, Yang Yang, Daiyong Huang, Lei Zhang, Rui Ran, Tian He, Yuanzhi Huang, Silas Villas‐Boas, Sergey Tumanov, Richard D. Cannon, Boris Novakovic, Richard Saffery, Yuhao Zhang, Xiaojing Dong, Ting‐Li Han

**Affiliations:** ^1^ Department of Obstetrics and Gynecology The Second Affiliated Hospital of Chongqing Medical University Chongqing China; ^2^ College of Food Science Southwest University Chongqing China; ^3^ Department of Pathology from College of Basic Medicine Chongqing Medical University Chongqing China; ^4^ Agilent Technology Guangzhou China; ^5^ Environmental Research and Innovation Department Luxembourg Institute of Science and Technology Esch‐sur‐Alzette Luxembourg; ^6^ Heart Research Institute The University of Sydney Newtown Australia; ^7^ Department of Oral Sciences Sir John Walsh Research Institute Faculty of Dentistry University of Otago Dunedin New Zealand; ^8^ Molecular Immunity Murdoch Children's Research Institute Royal Children's Hospital Melbourne Australia; ^9^ Chongqing Academy of Agricultural Sciences Chongqing China

**Keywords:** bile acids, gut microbiota, intrahepatic cholestasis of pregnancy, nanocellulose

## Abstract

Intrahepatic cholestasis of pregnancy (ICP) is a pregnancy‐specific disorder characterized by elevated maternal serum total bile acid (TBA) and increased risk of adverse fetal outcomes, yet current therapies and preventive strategies remain suboptimal. Here, we present a preclinical investigation of nanocellulose as a gut microbiota‐targeted dietary intervention to restore bile acid homeostasis. We demonstrate that nanocellulose, particularly cellulose nanofibers (CNF), administered prophylactically, attenuated maternal TBA and improved offspring survival in a rat ICP model by enriching probiotic microbial taxa while suppressing bile acid‐transforming bacteria. These microbial shifts promoted the accumulation of fecal primary bile acids and activation of gut–liver farnesoid X receptor (FXR) signaling. This cascade reduced intestinal reabsorption and hepatic synthesis of bile acids while promoting their excretion in feces. Collectively, pre‐gestational CNF effectively lowered systemic bile acid burden and mitigated the ICP phenotype. Our findings identify CNF as a biosafe, microbiota‐responsive dietary fiber with translational potential for early preventive management of bile acid‐related disorders during pregnancy.

## Graphical abstract

Nanocellulose, derived from microcrystalline cellulose (MCC) through sulfuric acid hydrolysis or mechanical grinding to produce CNC or CNF, was tested in a rat ICP model. Particularly, CNF improved gut microbiota composition, reduced secondary bile acid metabolism, and restored bile acid homeostasis through modulation of the gut–liver axis. This intervention effectively lowered systemic bile acid levels and enhanced offspring survival.

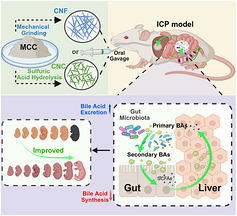

## 1 Introduction

Gestational hepatobiliary disorders represent a critical yet often overlooked aspect of maternal‐fetal health [1, 2]. Among these conditions, intrahepatic cholestasis of pregnancy (ICP) is the most common, emerging in late gestation and affecting up to 15% of pregnancies globally [3, 4]. Clinically, ICP is characterized by markedly elevated levels of maternal serum bile acids and liver transaminases, leading to profound adverse effects on both maternal and fetal health [5]. Of particular concern are the fetal complications, including meconium‐stained amniotic fluid, fetal distress, and even intrauterine death, ultimately contributing to increased perinatal mortality [5, 6]. Despite the grave threat these severe fetal complications pose, the primary treatment for ICP continues to be ursodeoxycholic acid (UDCA), a hydrophilic bile acid that is thought to reduce endogenous toxic bile acids in order to alleviate cholestasis [7]. However, its effectiveness in lowering serum bile acid levels and improving perinatal outcomes has been challenged by recent large‐scale randomized controlled trials and earlier population studies [8, 9].

The gut microbiota plays a crucial role in maintaining gut–liver metabolic homeostasis through its capacity to biotransform bile acids and regulate enterohepatic signaling [10]. Both the deconjugation of primary bile acids and the subsequent conversion of primary to secondary bile acids during enterohepatic circulation are critically dependent on specific microbial enzymatic functions. Hence, dysbiosis of the gut microbiota has been strongly associated with the pathogenesis of bile acid‐related diseases [11]. Clinically, previous studies have shown that the abundance of *Bacteroides*, *Lactobacillus*, and *Clostridium* species in the gut is significantly increased in patients with ICP [12, 13], accompanied by metabolic shifts in the gut microbiota that correlate with circulating bile acid levels and disease severity [13]. Similarly, rodent ICP models have demonstrated substantial shifts in gut microbial composition, accompanied by the increased susceptibility of offspring to adverse outcomes [14]. Importantly, recent mechanistic studies have identified specific microbial taxa in the gut as causal regulators of ICP progression. In particular, transferring the dominant bile salt hydrolase (BSH)‐producing bacterium *Bacteroides fragilis* from patients with severe ICP to pregnant mice successfully induced an ICP‐like phenotype. This effect was demonstrated to be mediated by BSH‐dependent inhibition of intestinal farnesoid X receptor (FXR) signaling, leading to derepression of hepatic bile acid synthesis, impaired biliary excretion, and pathological bile acid accumulation [15]. Together, these findings implicate a microbiota‐driven bile acid regulatory circuit between the gut and liver in ICP and support targeting the gut microbiome as a potential therapeutic approach.

Mounting evidence implicates the gut microbiota as a key contributor to the pathogenesis of ICP and as a promising dietary intervention strategy for diseases along the gut‐liver axis [16, 17]. Dietary fiber, broadly classified into soluble and insoluble types, is a key modulator of gut microbial structure and metabolic output [18]. Structurally and functionally, soluble fibers are readily fermented by gut microbes to generate bioactive metabolites such as short‐chain fatty acids (SCFAs), while insoluble fibers resist degradation, maintaining their physical structure and mechanically stimulate intestinal motility and preserve mucosal integrity [16, 18]. Recent advances in food nanotechnology have enabled the production of plant‐derived nanocellulose [19], a nanoscale insoluble dietary fiber characterized by a high aspect ratio, large specific surface area, and strong water‐holding capacity. Owing to these unique physicochemical features, nanocellulose exhibits gel‐like properties and enhanced interactions with bile acids, mucus, and microbial communities [20, 21]. Nanocellulose primarily consists of cellulose nanocrystals (CNC) and cellulose nanofibers (CNF) [22]. CNC, typically produced via acid hydrolysis from cellulose, exhibits a rigid rod‐like structure with high crystallinity and potential for surface functionalization. In contrast, CNF, generated through mechanical, enzymatic, or chemical fibrillation from cellulose, forms a flexible, entangled network with remarkable water absorption capacity, water retention, and flexibility [23]. Recent evidence suggests that nanocellulose can bind bile salts, lower serum lipid levels, and enrich commensal microbial taxa associated with SCFA production [24, 25]. These effects have been linked to improved gut barrier integrity and reduced intestinal inflammation in colonic disease models [26]. Furthermore, in *vitro* and in *vivo* studies have demonstrated that nanocellulose exhibits no cytotoxicity or adverse health effects when incorporated into food matrices [21]. However, despite extensive dietary fiber intervention studies in metabolic and inflammatory diseases, the translational potential and mechanistic rationale for nanocellulose to modulate the microbiota‐bile acid axis in pregnancy‐associated cholestasis remain undefined.

In this study, we developed a preclinical framework to investigate the potential of using two typical nanocelluloses (CNC and CNF) as an innovative dietary strategy for ICP management. Using in *vivo* experimentation coupled with integrative multi‐omics analysis, we present the first evidence that nanocellulose effectively alleviates ICP‐related phenotypes when administered preconceptionally. Mechanistically, we demonstrate that nanocellulose modulates the gut microbial ecosystem, thereby influencing the gut‐liver axis and reprogramming bile acid metabolism. These findings highlight the translational potential of microbiota‐responsive biomaterials as functional dietary interventions for preventing and managing bile acid‐related disorders, and support the application of nanocellulose as a safe, orally administrable dietary approach for non‐pharmacological management of ICP.

## 2 Results

### 2.1 Characterization of Nanocellulose

**FIGURE 1:**
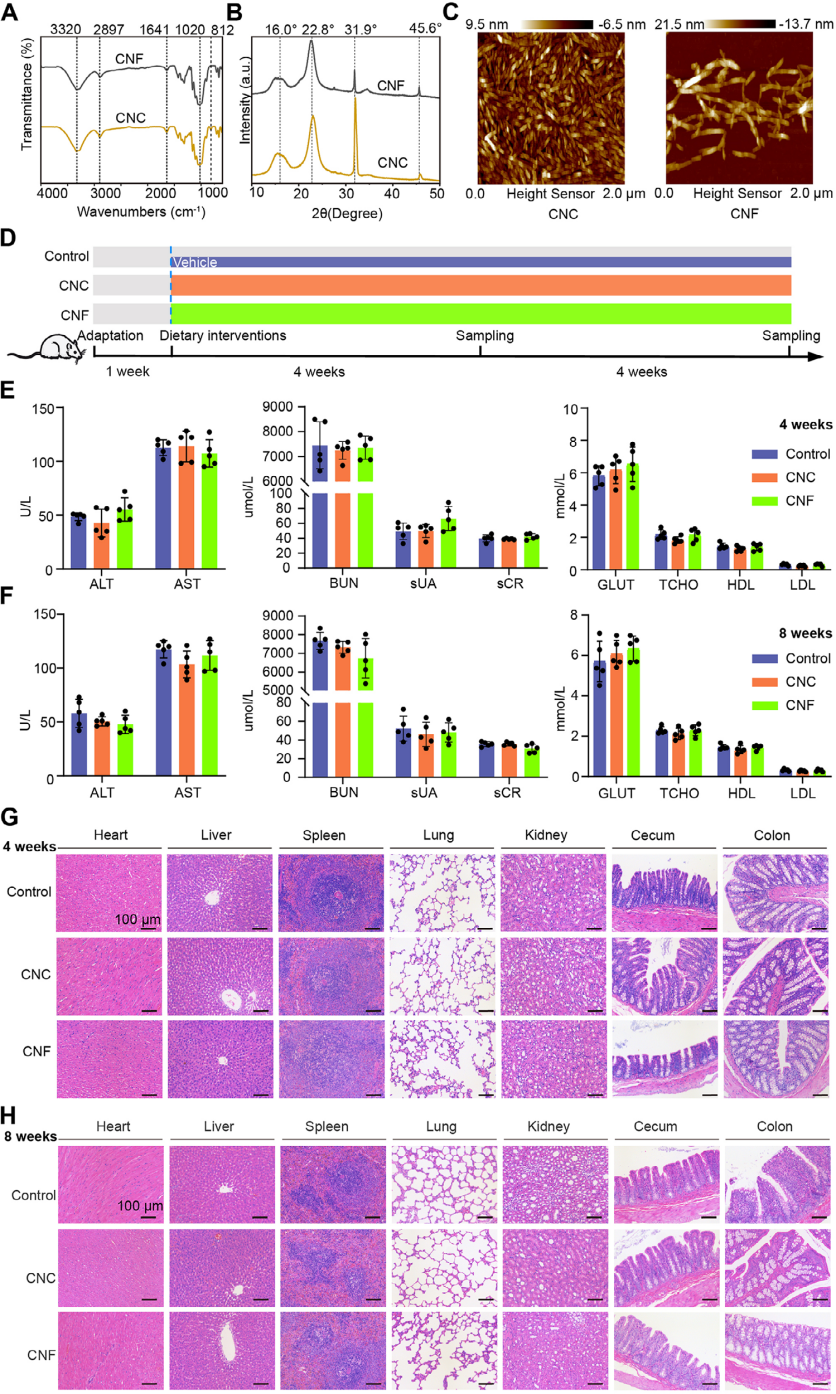
Characterization and long‐term safety assessment of nanocellulose. (A) FTIR spectra indicating the characteristic absorption peaks of CNC and CNF. (B) XRD patterns showing the crystalline diffraction peaks of CNC and CNF. (C) AFM images depicting the representative microstructures of CNC and CNF. (D) Schematic diagram of the experiment design for assessing the long‐term safety of orally administered nanocellulose in rats over 4 and 8 weeks. (E) and (F), Serum biochemical parameters [alanine aminotransferase (ALT), aspartate aminotransferase (AST), blood urea nitrogen (BUN), serum uric acid (sUA), serum creatinine (sCR), glucose (GLUT), and total cholesterol (TCHO), high‐density lipoprotein cholesterol (HDL), low‐density lipoprotein cholesterol (LDL)] after 4‐ and 8‐weeks administration, respectively (n = 5 per group). Data are presented as mean ± SEM. *p* values were determined by ordinary one‐way ANOVA with Tukey's post hoc test. (G) and (H), H&E staining of heart, liver, spleen, lung, kidney, cecum, and colon tissue samples, after 4‐ and 8‐weeks administration, respectively. Scale bar: 100 µm.

Fourier‐transform infrared spectroscopy (FTIR) analysis revealed similar absorption peaks for CNC and CNF (Figure 1A). Both exhibited a broad absorption peak at 3320 cm^−1^ corresponding to O─H stretching vibrations and peaks at 2897 cm^−1^ (C─H stretching), 1641 cm^−1^ (O─H bending), and pyranose ring ether bands at 1063 and 1052 cm^−1^. The peak at 892 cm^−1^ related to the β‐glycosidic bonds in cellulose. CNC exhibited a unique weak absorption peak at 815 cm^−1^ attributed to symmetric S─O─C stretching, due to sulfate ester groups introduced during sulfuric acid hydrolysis. These findings confirmed that CNC and CNF share a fundamental cellulose structure. X‐ray Diffraction (XRD) analysis further demonstrated that CNC and CNF maintained characteristic cellulose I diffraction peaks at 16.0°, 22.8°, and 31.9° (Figure 1B), with minimal differences in crystallinity (CNF: 70.92%; CNC: 71.05%), indicating neither the sulfuric acid hydrolysis nor the mechanical ball‐milling process significantly disrupted the crystalline structure of the cellulose. Atomic force microscopy (AFM) images revealed clear morphological differences between CNC and CNF (Figure 1C). CNC consisted of shorter, rod‐like structures (∼114 nm length, 28 nm diameter), whereas CNF exhibited longer fibrillar structures with partial aggregation (∼340 nm length, 52 nm diameter). These morphological differences were quantitatively analyzed using Image J. Taken together, these results confirmed that the preparation methods preserved the original crystalline structure of cellulose. However, CNC and CNF exhibited distinct microstructural morphologies, with CNC forming short, relatively rigid particles and CNF presenting as longer, more flexible nanofibrils.

### 2.2 Whole‐Gestation Maternal and Fetal Safety and 4‐8‐Week Long‐Term Outcomes Following Oral Nanocellulose Administration

The systemic safety of oral nanocellulose administration was first evaluated in non‐pregnant rats over an 8‐week period following daily gavage with CNC or CNF suspensions (Figure 1D). Serum biochemical markers were measured at 4 and 8 weeks to evaluate hepatic, renal, and metabolic function. Hepatic injury was evaluated using alanine aminotransferase (ALT) and aspartate aminotransferase (AST) assays. Renal function was assessed by blood urea nitrogen (BUN), serum uric acid (sUA), and serum creatinine (sCr) measurements. Metabolic status was evaluated by determining fasting glucose and lipid profiles, including those for total cholesterol (TCHO), high‐density lipoprotein cholesterol (HDL‐C), and low‐density lipoprotein cholesterol (LDL‐C). Histopathological examinations were also performed on major organs, including the heart, liver, spleen, lungs, kidneys, cecum, and rectum. No abnormal behavior or mortality was observed throughout the study period. Serum biochemical profiles remained comparable between nanocellulose‐treated and control groups, and no treatment‐associated alterations in organ morphology or tissue architecture were detected (Figures 1E–H).

To further evaluate maternal and fetal safety, we performed an additional full‐gestation oral dosing study in pregnant rats (Figure S1). Compared with normal pregnant controls, continuous nanocellulose administration throughout gestation resulted in no detectable alterations in maternal serum biochemical parameters, including liver and kidney function markers, lipid profiles, or cardiac enzyme indices (Figure S1A–E). Moreover, no significant differences were observed in maternal organ weights and fetal body weights, and histological examination revealed no overt abnormalities in major maternal organs or fetal tissues, including the heart, liver, and lung (Figure S1F–H).

### 2.3 Validation of the ICP Rat Model and Effects of Nanocellulose Intervention on ICP

**FIGURE 2:**
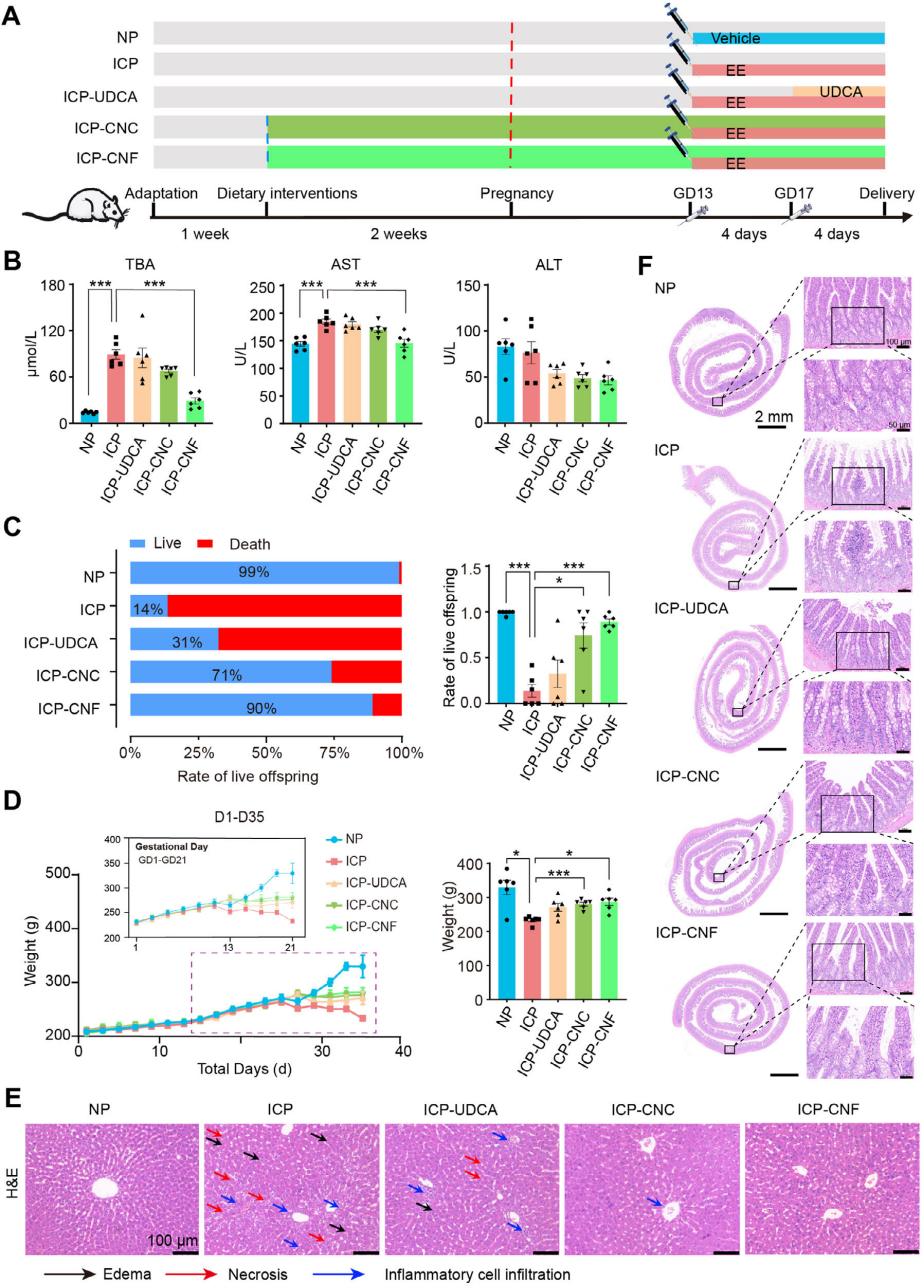
Effects of preconceptional nanocellulose dietary intervention on ICP rats. (A) Schematic diagram of the experiment design involving preconceptional nanocellulose dietary intervention, ICP model induction, and ursodeoxycholic acid (UDCA) treatment (n = 6 per group). (B) Serum levels of total bile acids (TBA), ALT, and AST. (C) Survival rates of offspring in the experimental groups. (D) Maternal body weight changes throughout the experiment period and gestation. Data in B‐D are presented as mean ± SEM. P values were calculated using one‐way ANOVA with Tukey's test or Welch's ANOVA with Games‐Howell's multiple comparisons test. (E) H&E staining of liver sections. Black arrows indicate hepatocellular edema; red arrows, necrosis; and blue arrows, inflammatory infiltration. Scale bar: 100 µm. (F) H&E staining of the distal ileum. Overview image: scale bar, 2 mm; magnified views: scale bars, 100 µm and 50 µm.*p < 0.05, **p < 0.01, ***p < 0.001.

In this study, we first conducted a timing‐of‐intervention analysis together with a dose‐ranging assessment to define the dosing regimen for nanocellulose intervention. A dose of 90 mg/kg/day initiated 14 days before pregnancy produced the most pronounced reduction in serum total bile acids (TBA) and liver injury markers (Figure S2). This regimen was therefore selected for the main in *vivo* experiments. Thirty outbred Sprague Dawley (SD) female rats were then randomly assigned to five groups (Figure 2A). Rats in the normal pregnancy group (NP) were given a standard diet, while rats in the ICP group were given a standard diet, CNC and CNF supplementary interventions prior to pregnancy, and continued using 17α‐ethinylestradiol (EE2) during pregnancy. The ICP group showed higher serum total bile acids (TBA) levels and lower offspring survival compared to the NP group, CNF exhibited a remarkable ability to ameliorate the ICP phenotype, including a significant reduction in TBA levels and an improvement in offspring survival rates (Figure 2B,C and Table S1). These findings indicate that dietary nanocellulose supplementation improved ICP‐related serum biomarker levels, with CNF demonstrating superior therapeutic potential to CNC and UDCA.

ICP affected offspring survival rates (Figure 2C). Compared to the ICP group with only 14% offspring survival, UDCA intervention resulted in a modest improvement of 17%, while CNC and CNF supplementation significantly enhanced survival rates by 57% and 76%, respectively (Figure 2C). Increasing the UDCA dose to 50 mg/kg/day did not result in a statistically significant improvement in serum TBA, liver injury markers, or live birth rate relative to the 25 mg/kg/day regimen (Figure S3). In terms of body weight trajectories, the most pronounced disparity was observed between the ICP and NP groups. Notably, nanocellulose supplementation counteracted the abnormal weight fluctuations and reduction associated with ICP, restoring a growth pattern comparable to that of the NP group (Figure 2D). Histological analysis of liver tissue (Figure 2E) with H&E staining indicated that the NP group displayed structurally intact liver lobules. In contrast, the ICP group exhibited disorganized hepatocyte arrangements, extensive inflammatory infiltration, edema, intercellular space widening, and localized hepatic necrosis (Figure 2E). These pathological alterations persisted in the ICP‐UDCA group, albeit to a lesser extent, but were markedly improved in the ICP‐CNC group. Notably, the ICP‐CNF group exhibited the most pronounced improvement, with structures closely resembling the NP group. Histological examination of ileal tissue (Figure 2F) revealed that the ICP group exhibited severe villous atrophy, extensive inflammatory infiltration, and crypt shallowing compared to the NP group. Although crypt depth was partially recovered in the ICP‐UDCA group, inflammatory infiltration remained prominent. The ICP‐CNC group demonstrated substantial histological improvement and the ICP‐CNF group exhibited the most comprehensive recovery, with the villous morphology closely resembling that of the NP group (Figure 2F).

Given that fetal outcome is a primary clinical concern in ICP, fetal growth and placental pathology were further evaluated. Histological examination of placental tissues revealed prominent inflammatory cell infiltration and intracellular edema of trophoblasts in the ICP group (Figure S4A). These pathological alterations were markedly attenuated in the nanocellulose‐treated groups, particularly in the CNF group. Compared with the ICP group, fetal body weight was significantly increased in both the ICP‐CNC and ICP‐CNF groups, with the most pronounced improvement observed in the CNF‐treated group (Figure S4B).

Collectively, these findings confirm the successful establishment of the ICP rat model and highlight the protective potential of nanocellulose intervention, particularly CNF.

### 2.4 Impact of Nanocellulose on Gut Microbiota Composition

**FIGURE 3:**
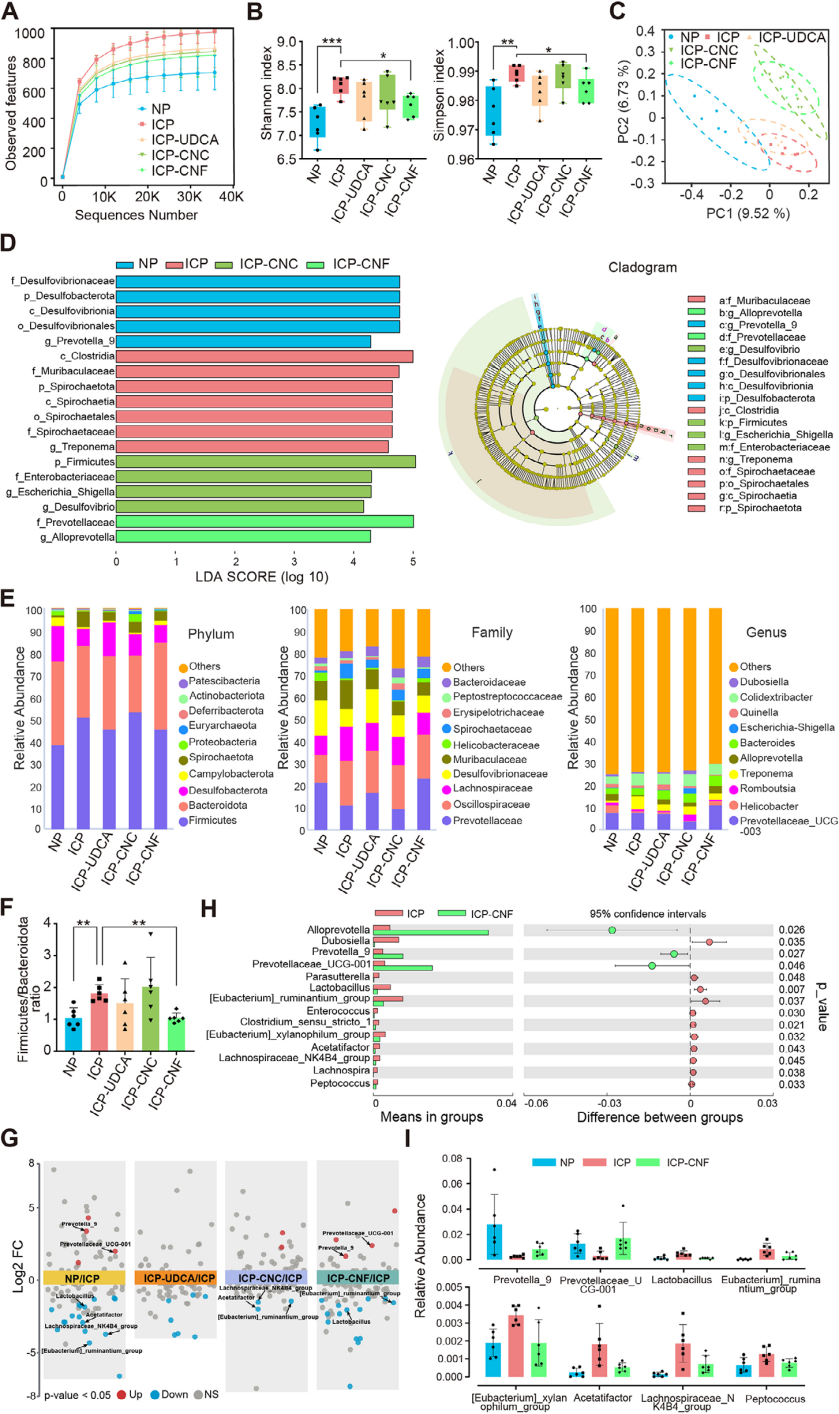
Nanocellulose modulates gut microbiota composition in ICP rats. (A) Observed operational taxonomic units (OTUs) reflecting alpha diversity in the groups. (B) Shannon and Simpson diversity indices. *P* values were determined using the Kruskal‐Wallis test. (C) Principal coordinate analysis (PCoA) illustrating group‐wise differences in beta diversity. (D) LEfSe cladogram identifying significantly enriched taxa (LDA score > 4, p < 0.05). Taxa enriched in specific groups are shown in color; non‐significant taxa are shown in yellow. (E) Relative abundances of dominant taxa at the phylum, family, and genus levels. (F) Firmicutes/Bacteroidota ratio in the groups. Data are presented as mean ± SEM, *p* values were determined by Welch ANOVA with Games‐Howell's multiple comparisons test. (G) Genera significantly altered in the NP and intervention groups compared to the ICP group. Red dots indicate an increase, while blue dots indicate a decrease in relative abundance compared to the ICP group. (H) Genera differentially enriched between the ICP and ICP‐CNF groups. (I) Distribution of representative genera among the NP, ICP, and ICP‐CNF groups. *p < 0.05, **p < 0.01, ***p < 0.001.

Dietary fiber can influence the composition and diversity of gut microbiota. To investigate the differences in gut microbiota, 16S rRNA gene sequencing was performed on fecal samples from the experimental groups. The number of operational taxonomic units (OTUs) was highest in the ICP group and lowest in the NP group, indicating increased richness under cholestatic conditions that is consistent with microbiota dysbiosis. Following intervention, the OTU profiles of the ICP‐CNC and ICP‐CNF groups shifted toward those of the NP group (Figure 3A). Similarly, both the Shannon index and the Simpson index (Simpson's Index of Diversity, 1 – D) were increased in the ICP group, reflecting higher α‐diversity and increased evenness with reduced dominance of major taxa. CNF intervention partially restored these indices toward NP levels (Figure 3B). Principal Coordinate Analysis (PCoA) was performed to visualize differences in beta‐diversity between the samples, with closer distances indicating more similar species compositions (Figure 3C). The PCoA results demonstrated high within‐group similarity in the fecal microbiota across all five groups, with clear distinctions observed between the ICP and NP groups. Both the ICP‐CNC and ICP‐CNF groups clustered together, indicating a similar β‐diversity profile. Notably, a degree of overlap between the ICP and ICP‐UDCA groups was observed, which may suggest suboptimal microbiome compositions following UDCA treatment.

The gut microbiota structure at the phylum to genus levels was further investigated and is illustrated in the LEfSe cladogram (Figure 3D). Significant variations in bacterial composition were observed between the groups (LDA > 4). Specifically, the NP group exhibited a higher relative abundance of the phylum *Desulfobacterota* and genus *Prevotella_9*. Conversely, the ICP group was enriched in the phylum *Spirochaetota* and genus *Treponema*. The ICP‐CNC group showed an increased abundance of the phylum *Firmicutes* and genera *Escherichia‐Shigella* and *Desulfovibrio*. In contrast, the ICP‐CNF group was characterized by a higher prevalence of the family *Prevotellaceae* and genus *Alloprevotella*. Notably, no significant differences in bacterial taxa were detected in the ICP‐UDCA group. Furthermore, the gut microbiota structure at phylum, family, and genus levels was further investigated (Figure 3E). At the phylum level, compared to the NP group, the ICP group showed an increased relative abundance of *Firmicutes*, *Spirochaetota*, and a decreased abundance of *Desulfobacterota*, *Campylobacterota*, *Proteobacteria*, and *Euryarchaeota*. In addition, the elevated *Firmicutes/Bacteroidota* ratio observed in the ICP group, compared to the NP group, further indicated disruption of the gut microbiome (Figure 3F). At the genus level, compared to the NP group, the ICP group had a significantly reduced relative abundance of probiotic bacteria such as *Helicobacter*, *Romboutsia*, and *Alloprevotella*, along with an increase in the butyrate‐producing *Treponema* and the pathogenic genus *Colidextribacter*. Building on the observed microbial compositional and abundance shifts in the ICP group, the gut microbiota profile of the ICP‐UDCA group remained largely unchanged. In contrast, nanocellulose intervention, particularly in the ICP‐CNF group, significantly restored the overall gut microbial composition. This normalization was evident across multiple taxonomic levels, including the phylum level (e.g., the *Firmicutes*/*Bacteroidota* ratio), as well as the family and genus levels, rendering the microbial profile more similar to that of the NP group.

Comparative analysis revealed that the NP and ICP‐CNF groups exhibited a greater number of bacterial taxa with similar directional shifts – either increased or decreased – than the ICP group. Specifically, *Prevotella_9* and *Prevotellaceae_UCG‐001* were significantly enriched in both the NP and ICP‐CNF groups, while *Lactobacillus*, *Eubacterium ruminantium group*, *Eubacterium xylanophilum group*, *Acetatifactor*, *Lachnospiraceae NK4B4 group*, and *Peptococcus* were markedly reduced (Figure 3G‐I and Figure S5A). In contrast, differential microbiota analysis between the ICP‐UDCA and ICP groups showed that *Parasutterella*, *Alistipes*, and *Corynebacterium* were significantly more abundant in the ICP group than in the ICP‐UDCA group (Figure S5B). Similarly, in the ICP‐CNC group, *Desulfovibrio*, *Bilophila*, and *Ligilactobacillus* were significantly enriched compared to the ICP group, whereas the *Eubacterium ruminantium group*, *Acetatifactor*, and *Lachnospiraceae NK4B4 group* were more abundant in the ICP group than in the ICP‐CNC group (Figure S5C). These findings reinforce the association between ICP and gut microbiota dysbiosis while highlighting the potential of nanocellulose, particularly CNF, in modulating microbial composition and restoring gut homeostasis in ICP.

### 2.5 Influence of Nanocellulose on Liver–Gut Bile Acid Profiles

**FIGURE 4:**
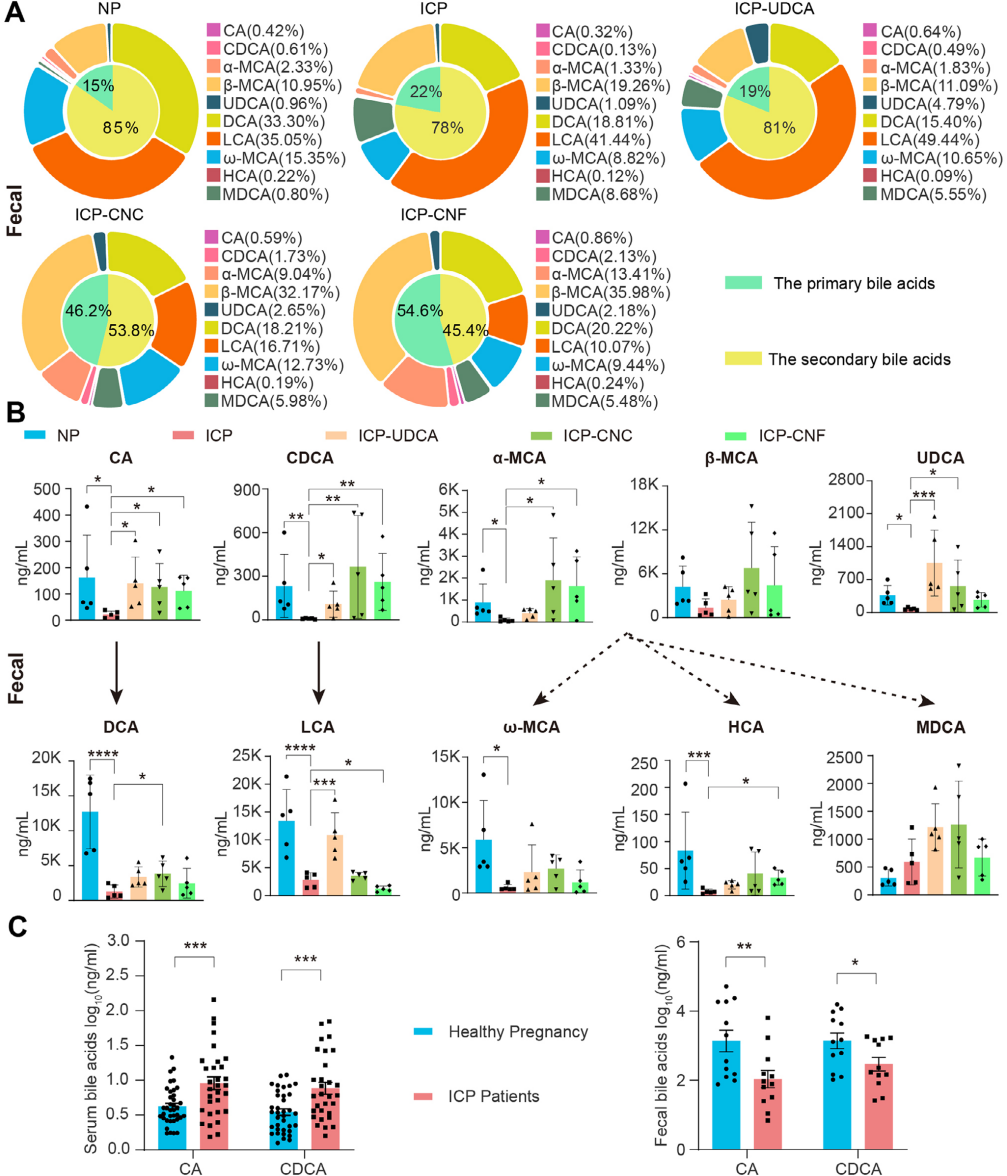
Nanocellulose alters fecal bile acid profiles along the gut‐liver axis. (A) Composition of bile acid subtypes in fecal samples from each group (stacked segments; normalized to 100%). (B) Distribution of representative bile acids with differential abundancies in fecal samples. (C) Fecal and serum levels of CA and CDCA in healthy pregnant women and patients with clinical ICP. Data are presented as mean ± SEM. *P* values were determined by one‐way ANOVA with Tukey's post hoc test or Welch's ANOVA followed by Games‐Howell correction. K indicates ×1000. *p < 0.05, **p < 0.01, ***p < 0.001, ****p < 0.0001.

To further investigate bile acid metabolism, bile acid profiles in cecal contents, liver, and blood samples were analyzed using a highly sensitive LC‐MS/MS method. The bile acids were systematically categorized into primary and secondary bile acids, along with their respective conjugated forms. Both qualitative (Figure 4A and Figure S6A,C) and quantitative (Figure 4B and Figure S6B,D) analyses were performed to assess proportional and compositional differences between groups.

Notably, primary bile acids were predominant in liver and serum samples, whereas secondary bile acids were more abundant in fecal samples. A key observation was the increased proportion of primary bile acids in the feces, liver, and serum of the ICP‐CNC and ICP‐CNF groups compared to the ICP and ICP‐UDCA groups (Figure 4A and Figure S6A,C). Specifically, in fecal samples (Figure 4A), the proportions of cholic acid (CA) and chenodeoxycholic acid (CDCA) were lower in the ICP group compared to the NP, ICP‐UDCA, ICP‐CNC, and ICP‐CNF groups. In contrast, lithocholic acid (LCA) was elevated in the ICP and ICP‐UDCA groups relative to the ICP‐CNF group. In liver tissue (Figure S6A), the proportions of α‐muricholic acid (α‐MCA) and β‐muricholic acid (β‐MCA) were higher in the ICP and ICP‐UDCA groups than in the NP, ICP‐CNC, and ICP‐CNF groups. In serum samples (Figure S6C), the proportions of β‐MCA, glycochenodeoxycholic acid (GCDCA), and tauro‐β‐muricholic acid (T‐β‐MCA) were lower in the NP, ICP‐CNC, and ICP‐CNF groups compared to the ICP and ICP‐UDCA groups. These findings highlight a distinct alteration in bile acid profiles between the NP and ICP groups. Importantly, nanocellulose intervention, particularly CNF, altered fecal bile acid subclass composition (Figure 4A), characterized by an increased proportional contribution of primary bile acids, suggesting enhanced luminal retention and fecal excretion of bile acids.

Quantitative measurements further demonstrated corresponding changes in absolute bile acid concentrations across fecal, hepatic, and serum compartments (Figure 4B and Figure S6B,D). In fecal samples (Figure 4B), compared to the ICP group, the NP, ICP‐CNC, and ICP‐CNF groups showed elevated levels of CA, CDCA, and α‐MCA, with a notable reduction in LCA observed in the ICP‐CNF group. In liver tissue (Figure S6B), the ICP‐CNF group exhibited a significant decrease in CDCA, α‐MCA, β‐MCA, GCDCA, T‐α‐MCA, T‐β‐MCA, TUDCA, and GUDCA compared to the ICP group, along with an increase in TCDCA. A similar pattern was observed in serum samples (Figure S6D), where the NP and ICP‐CNF groups displayed a marked reduction in CA, CDCA, GCDCA, α‐MCA, T‐α‐MCA, β‐MCA, T‐β‐MCA, UDCA, GUDCA, TDCA, and ω‐MCA compared to the ICP group. These findings suggest that nanocellulose, particularly CNF, effectively modulated the composition and concentration of bile acids, thereby restoring a more balanced bile acid profile, reducing serum bile acid accumulation, and potentially alleviating ICP‐related metabolic disturbances.

### 2.6 CNF‐Induced Microbiota Remodeling Suppresses CDCA‐to‐LCA Conversion

To further delineate how nanocellulose‐induced microbiota remodeling reshapes bile acid metabolism, we evaluated microbial bile acid‐transforming capacities, focusing on BSH‐mediated deconjugation and 7α‐dehydroxylation, the major route to secondary bile acid production. Fecal suspensions prepared from ICP, ICP‐CNC, and ICP‐CNF groups were first subjected to BSH activity assays (Figure S7A). No significant differences in BSH activity were observed among the three groups. Notably, in *vivo* CNF treatment markedly attenuated CDCA‐to‐LCA conversion (Figure S7B), whereas DCA‐to‐CA conversion showed no significant changes (Figure S7C). We therefore focused on the microbial 7α‐dehydroxylation pathway, a multistep biochemical process essential for secondary bile acid formation. To validate, functionally, whether nanocellulose‐induced microbial remodeling directly constrains this process, fecal bacterial suspensions derived from the three groups were incubated with the primary bile acid CDCA. Notably, bacterial communities derived from the ICP‐CNF group exhibited a significantly attenuated in *vitro* capacity for CDCA‐to‐LCA conversion (Figure S7D), indicative of a constraint on microbial 7α‐dehydroxylation. These findings demonstrate that CNF selectively attenuates microbiota‐mediated secondary bile acid formation by constraining the microbial 7α‐dehydroxylation pathway, thereby promoting the accumulation of CDCA.

### 2.7 Bile Acid Profile Alterations in Clinical ICP

To confirm alterations in bile acid subtypes associated with ICP, fecal and serum samples were collected from 31 patients diagnosed with ICP and 37 gestational age‐matched healthy pregnant participants. In patients with clinical ICP, fecal CA and CDCA levels were significantly reduced, whereas their serum concentrations were markedly elevated compared to healthy pregnant women (Figure 4C). These alterations closely resembled the bile acid profiles observed in ICP rats, particularly relative to the NP and ICP‐CNF groups.

### 2.8 Effects of Nanocellulose on Gut–Liver Metabolic Reprograming

**FIGURE 5:**
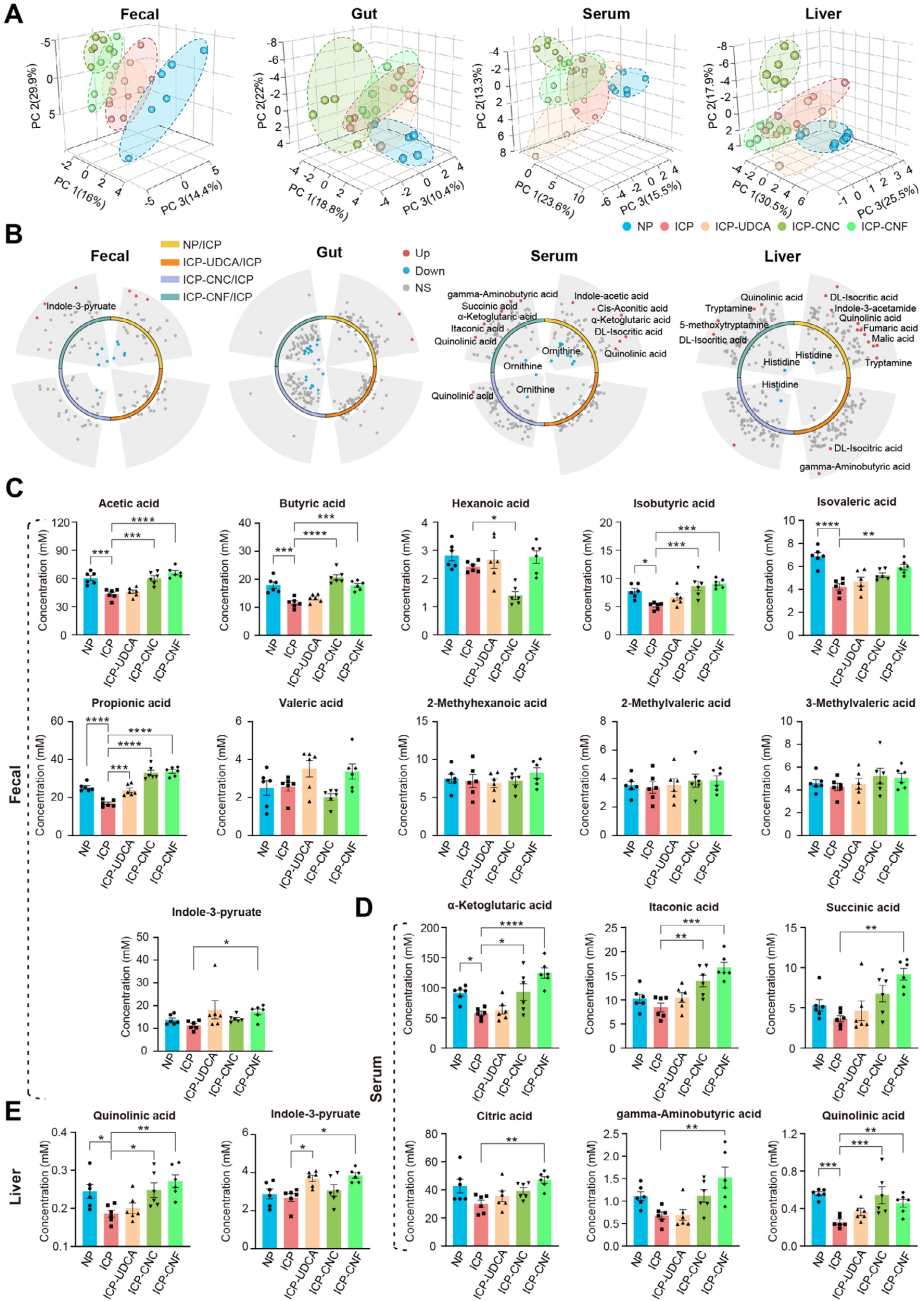
Nanocellulose affects metabolic signatures across the gut‐liver axis. (A) Partial least squares discriminant analysis (PLS‐DA) of metabolite profiles in feces, intestinal wall, serum, and liver samples across groups. (B) Volcano plots showing significantly altered metabolites (*p* < 0.05, |log_2_ fold change| > 1.0) among the five groups in feces, gut, serum, and liver. Red dots indicate upregulated metabolites, while blue dots indicate downregulated metabolites in response to the ICP group. (C) Levels of short‐chain fatty acids (SCFAs) and representative indole‐derived metabolites in fecal samples. (D) Serum concentrations of representative tricarboxylic acid (TCA) cycle intermediates and tryptophan metabolites. (E) Concentrations of representative tryptophan‐derived metabolites in liver tissue. Data are presented as mean ± SEM. *P* values were determined by one‐way ANOVA with Tukey's multiple comparisons test. *p < 0.05, **p < 0.01, ***p < 0.001, ****p < 0.0001.

Beyond investigating the effects of nanocellulose intervention on bile acid metabolism, the study evaluated its impact on the remodeling of metabolite pathways associated with tryptophan metabolism, the tricarboxylic acid (TCA) cycle, and lipid metabolism (Figure 5 and Figures S8 and S10).

A total of 110 metabolites were identified across the fecal, gut, serum, and liver samples. Three‐dimensional partial least squares discriminant analysis (PLS‐DA) revealed distinct separations in the metabolite profiles of the five experimental groups, while maintaining high intra‐group reproducibility across all sample types (Figure 5A and Figure S8A). The Venn diagram analysis revealed that with nanocellulose intervention, the ICP‐CNC and ICP‐CNF groups exhibited shared differentially abundant metabolites in fecal (1), gut (37), serum (10), and liver (7) samples (Figure S8B,C). Moreover, they also shared differentially abundant metabolites with the NP group. In contrast, no differentially abundant metabolites were detected in the fecal, gut, or serum samples of the ICP‐UDCA group when compared to the ICP group. Upset plot analysis revealed that the ICP‐CNC group had one unique metabolite in the gut, while the ICP‐CNF group had unique metabolites in fecal, gut, serum, and liver samples. In addition, several differentially abundant metabolites, including indole‐3‐propionic acid and tryptophan, were shared among the NP, ICP‐CNC, and ICP‐CNF groups across different samples. These patterns indicate that nanocellulose intervention induces more pronounced metabolic alterations in serum and the liver than in feces and the gut.

Furthermore, significant alterations in metabolite concentrations between feces, gut, serum, and liver samples were identified and distinguished using volcano plots (Figure 5B) and heatmaps (Figures S8D and S9A). Overall, only a few significant alterations were observed in fecal samples. Most metabolites showed reduced concentrations in the gut, whereas serum and liver samples exhibited higher metabolite concentrations in the nanocellulose intervention groups than in the ICP group. In particular, the fecal metabolome exhibited markedly higher concentrations of microbial‐derived indole‐3‐pyruvate and SCFAs, including acetic acid, butyric acid, isobutyric acid, isovaleric acid, and propionic acid, in the nanocellulose‐treated groups than in the ICP group (Figure 5C). In the serum metabolome, significant elevations were observed in TCA cycle intermediates and derivatives, including α‐ketoglutaric acid, itaconic acid, succinic acid, citric acid, malic acid, and fumaric acid, as well as tryptophan derivatives such as gamma‐aminobutyric acid, quinolinic acid, and picolinic acid in the nanocellulose intervention group compared to the ICP group (Figure 5D and Figure S9B). In the liver metabolome, the levels of quinolinic acid and indole‐3‐pyruvate were markedly increased in the nanocellulose intervention groups, particularly in the ICP‐CNF group, compared to the ICP group (Figure 5E). These findings suggest that nanocellulose intervention promotes the normalization of gut‐liver axis metabolite profiles under ICP conditions, with a more pronounced effect in the ICP‐CNF group. The KEGG metabolic framework was used to annotate pathways associated with the differential abundancies of metabolites between the ICP and ICP‐CNF groups. Comparative pathway enrichment analysis (Figure S9C) revealed that the fecal metabolome of the ICP‐CNF group had five significantly upregulated pathways, most notably *bile acid biosynthesis*, and six downregulated pathways, including *unsaturated fatty acid biosynthesis*. In serum, ten pathways were upregulated (e.g., *oxidative phosphorylation*, *glutathione*, *pyruvate*, and *butyrate metabolism*), while three were downregulated, prominently *indole alkaloid biosynthesis* and *oxytocin signaling*; 19 biologically relevant metabolites were annotated within these pathways (Figure S9D).

Thus, these findings demonstrate that nanocellulose intervention, particularly CNF, can significantly reshape metabolite profiles in feces, serum, and liver, promoting a metabolic shift from the ICP‐associated state toward one resembling the NP group.

### 2.9 Correlation Between Gut Microbiota, ICP‐Related characteristics, Bile Acids, TCA and Tryptophan Derivatives

CNF intervention significantly altered the gut microbiota, which was strongly associated with key clinical and metabolic features. Beneficial taxa such as *Alloprevotella*, *Prevotellaceae_UCG‐001*, and *Prevotella_9* were significantly correlated with improved liver function (as indicated by lower TBA and AST levels), increased concentrations of SCFAs, tryptophan metabolites, and TCA cycle intermediates, as well as lower bile acids in both serum and liver. Conversely, bacterial genera such as *Lactobacillus*, *Clostridium sensu stricto 1*, and *Acetatifactor* exhibited opposing associations, with higher serum and liver bile acid levels, as well as reduced metabolic outcomes. These correlations suggest CNF promotes a gut microbiota profile linked to favorable metabolic and clinical outcomes across the gut‐liver axis (Figure S10).

### 2.10 Microbiota Transfer and FXR Antagonism Confirm CNF‐Mediated Protection in ICP

**FIGURE 6:**
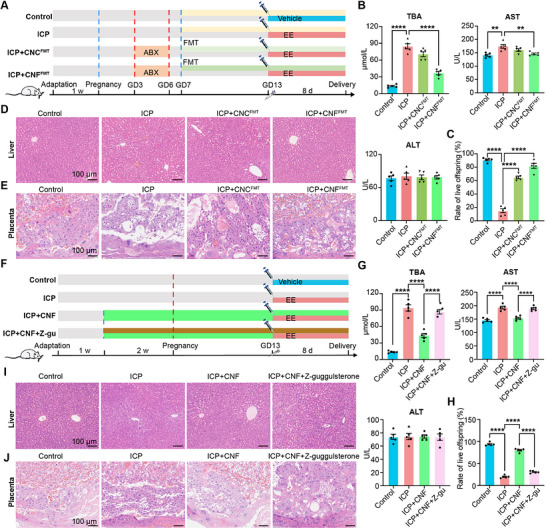
Microbiota transfer and FXR antagonism validate the protective effects of CNF in ICP. (A) Schematic illustration of the fecal microbiota transplantation (FMT) experimental design (n = 5 per group). (B) Serum levels of total bile acids (TBA), aspartate aminotransferase (AST), and alanine aminotransferase (ALT). (C) Live birth rate in each group. (D) Representative H&E‐stained liver sections. (E) Representative H&E‐stained placental sections. (F) Schematic illustration of the FXR antagonist experiment using Z‐guggulsterone (Z‐gu) (n = 5 per group). (G) Serum levels of TBA, AST, and ALT. (H) Live birth rate. (I) Representative H&E‐stained liver sections. (J) Representative H&E‐stained placental sections. Data in (B), (C), (G), and (H) are presented as mean ± SEM. Statistical significance was determined by one‐way ANOVA followed by Tukey's multiple‐comparisons test. Scale bar, 100 µm. *p < 0.05, **p < 0.01, ***p < 0.001.

Building on the pronounced protective effects of nanocellulose on ICP‐related phenotypes, we next performed fecal microbiota transplantation (FMT) to determine whether gut microbiota alterations are sufficient to transmit these benefits. Fecal microbiota collected from ICP‐CNC and ICP‐CNF donor rats were transplanted into pregnant ICP recipient rats (Figure 6A). Compared with ICP, rats receiving microbiota from the ICP‐CNF group exhibited significantly reduced serum TBA and AST levels, accompanied by a marked increase in live birth rate and substantial improvement in hepatic and placenta histopathology (Figure 6B–E). Transplantation of microbiota from the ICP‐CNC group also conferred partial protection, consistent with its more modest phenotypic effects observed in donor animals. These results demonstrate that gut microbiota remodeling induced by nanocellulose, particularly CNF, is sufficient to ameliorate ICP‐related phenotypes, supporting a causal contribution of the microbiota to the protective effects of CNF.

We next examined whether FXR signaling is required for CNF‐mediated protection. The FXR antagonist Z‐guggulsterone was therefore administered concomitantly with CNF treatment (Figure 6F). Pharmacological inhibition of FXR markedly attenuated the CNF‐induced reduction in serum TBA and AST levels (Figure 6G). Moreover, the beneficial effects of CNF on fetal survival and hepatic and placenta histopathological alterations were largely abolished in the presence of Z‐guggulsterone (Figure 6H–J). These findings indicate that FXR signaling is required for the protective effects of CNF against ICP‐related phenotypes.

### 2.11 Nanocellulose‐Mediated Regulation of Key Bile Acid Metabolic Enzymes and Transporters in the ICP Gut–Liver Axis

**FIGURE 7:**
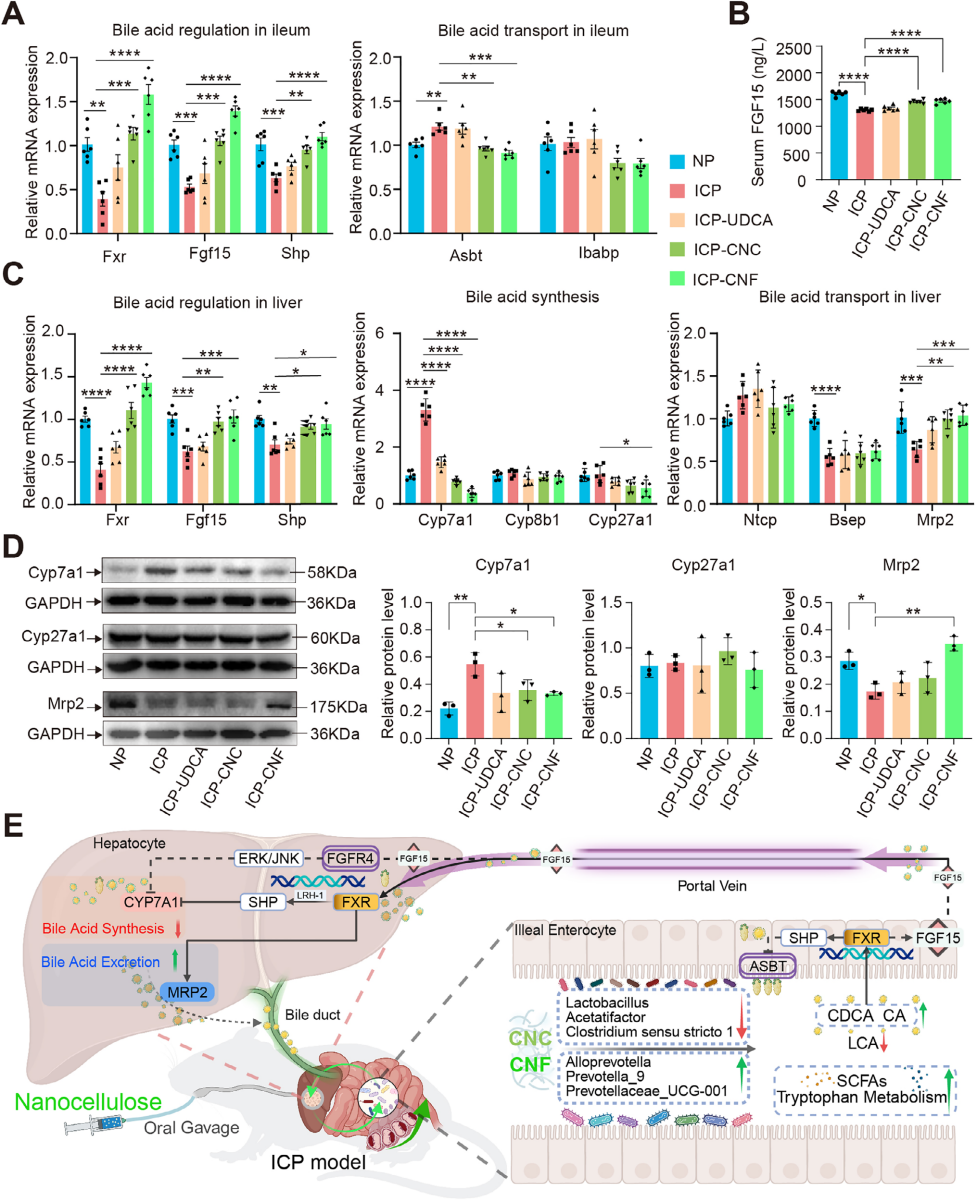
Nanocellulose modulates gut‐liver bile acid metabolism‐related gene and protein expression. (A) Expression levels of bile acid‐related regulatory and transporter genes in the distal ileum of rats. (B) Serum levels of fibroblast growth factor 15 (FGF15). (C) Hepatic expression of genes involved in bile acid regulation, synthesis, and excretion. (D) Protein expression levels of hepatic Cyp7a1, Cyp27a1, and Mrp2. Data in A–D are presented as mean ± SEM. P values were determined by one‐way ANOVA with Tukey's post hoc test or Welch's ANOVA followed by Games‐Howell's test. (E) Schematic diagram of the mechanisms by which nanocellulose improves ICP through modulation of the gut microbiota‐bile acid‐intestinal‐liver FXR signaling pathway. *p < 0.05, **p < 0.01, ***p < 0.001, ****p < 0.0001.

To elucidate the molecular mechanisms underlying the beneficial effects of nanocellulose, particularly CNF, on ICP we examined the expression of key components of the bile acid regulatory network. mRNA levels of genes involved in bile acid signaling (*Fxr*, *Fgf15*, *Shp*), transport (*Asbt*, *Ibabp*, *Ntcp*, *Bsep*, and *Mrp2*), and synthesis (*Cyp7a1*, *Cyp27a1*, and *Cyp8b1*) were quantified by qPCR in both terminal ileum and liver tissues. In parallel, the protein expression of the major bile acid metabolic enzymes (Cyp7a1 and Cyp27a1) and transporters (Mrp2) was assessed by Western blotting, and circulating Fgf15 levels were measured in serum (Figure 7).

Specifically, *Fxr*, *Fgf15*, and *Shp* mRNA expression was significantly lower in ICP rats than in the NP group in the ileum tissue (Figure 7A). No significant change was observed in the ICP‐UDCA group; however, *Fxr*, *Fgf15*, *Shp* expression was notably elevated in the ICP‐CNC and ICP‐CNF groups compared to the ICP group. Further examination of bile acid transport genes and proteins in the terminal ileum revealed that Asbt expression was significantly higher in the ICP group than in the ICP‐CNF group (Figure S11), with no significant difference compared to the ICP‐UDCA group. In contrast, *Ibabp* gene expression showed no significant differences between groups. Consistent with these findings, serum FGF15 levels (Figure 7B) were significantly lower in the ICP group than in the NP, ICP‐CNC, and ICP‐CNF groups. In the liver, the trends of *Fxr*, *Fgf15*, and *Shp* mRNA expression in the different groups were consistent with those observed in the ileum (Figure 7C). Regarding enzymes involved in bile acid synthesis in liver samples, key enzymes from the classical and alternative pathways, Cyp7a1 and Cyp27a1, were analyzed further. *Cyp7a1* mRNA levels were significantly higher in the ICP group than in the NP group, with marked reductions observed following CNC and CNF interventions. Interestingly, while there was no difference in *Cyp27a1* expression between the NP and ICP groups, its expression was significantly lower in the ICP‐CNF group than in the ICP group. Meanwhile, *Cyp8b1* expression remained unchanged in all groups. Regarding bile acid transporters in the liver, there was significantly lower mRNA expression for Mrp2, a crucial enzyme for bile acid excretion, in the ICP group than in the ICP‐CNC and ICP‐CNF groups, with no significant difference from the ICP‐UDCA group. In addition, *Bsep* expression was lower in the ICP group than in the NP group, with no changes observed after UDCA or nanocellulose interventions, while *Ntcp* mRNA levels remained unchanged in all groups. Furthermore, for genes that displayed significant changes in expression, the corresponding protein levels, including Cyp7a1, Cyp27a1, and Mrp2, were measured using Western blot analysis (Figure 7D). Cyp7a1 protein levels were remarkably elevated in the ICP group compared to the NP group, but were reduced following CNC and CNF interventions. Meanwhile, Cyp27a1 protein levels remained unchanged in all groups. Moreover, Mrp2 protein expression was lower in the ICP group than in the NP group but was significantly higher in the ICP‐CNF group than in the ICP group, paralleling the mRNA results.

Considering the observed modulation of bile acid homeostasis along the gut‐liver FXR axis by CNF, we then performed in *vitro* experiments using intestinal epithelial and hepatic cell models to validate the CDCA‐FXR‐FGF19 Signaling Axis at the cellular level. Caco‐2 cells were treated with CDCA, with the synthetic FXR agonist GW4064 included as a positive control (Figure S12A–C). CDCA treatment significantly activated FXR signaling in Caco‐2 cells, as evidenced by increased expression of FXR target genes and elevated FGF19 secretion into the culture supernatant (Figure S12A,C). In parallel, Western blot analysis revealed a marked reduction in the expression of the bile acid reabsorption transporter ASBT following CDCA stimulation (Figure S12B). To further assess gut‐liver signaling, conditioned medium from CDCA‐treated Caco‐2 cells was applied to hepatocytes. This treatment led to significant activation of hepatic FXR signaling (Figure S12D), accompanied by suppression of the bile acid synthesis enzyme CYP7A1 and upregulation of the bile acid efflux transporter MRP2 at both the mRNA and protein levels (Figure S12E). Collectively, these results demonstrate that CDCA‐mediated activation of intestinal FXR promotes FGF19 signaling, which in turn regulates hepatic bile acid synthesis and transport pathways, providing direct cellular evidence for the gut‐liver FXR axis proposed in this study.

In summary, nanocellulose, particularly CNF, may modulate bile acid metabolism in ICP via FXR activation and downstream targets, contributing to the restoration of bile acid homeostasis and the alleviation of ICP symptoms.

## 3 Discussion

ICP is a pregnancy‐specific disorder characterized by elevated maternal serum TBA levels and is associated with adverse perinatal outcomes. Effective therapeutic interventions are currently lacking, and the underlying mechanisms remain unclear. To investigate potential strategies for mitigating ICP‐related complications, we prepared plant‐derived nanocellulose and applied it as a pre‐pregnancy intervention in a rat ICP model. We found that nanocellulose exhibited excellent biocompatibility and safety, significantly reduced serum TBA levels, and improved offspring survival rates, with CNF showing particularly pronounced beneficial effects. Furthermore, nanocellulose intervention provided additional benefits by modulating gut microbiota composition and activating the gut‐liver FXR pathway. This activation reduced terminal ileum bile acid reabsorption, suppressed hepatic bile acid synthesis, and enhanced bile acid excretion into the bile duct. Together, these findings highlight the potential of nanocellulose supplementation as a preventive strategy for ICP.

Our study demonstrated the superiority of CNF over CNC in the therapeutic effect on ICP rats. Both CNC and CNF retained the characteristic cellulose I crystalline structure, as confirmed by FTIR and XRD analyses (Figure 1A,B) [27, 28, 29, 30]. However, despite sharing an identical chemical composition and crystalline framework, their distinct nanoscale architectures confer fundamentally different physicochemical and biological properties. In the present study, CNF was prepared by ball milling using NaOH as the milling medium, resulting in an overall increase in crystallinity [31]. This structural feature may contribute to the enhanced functional performance of CNF. Consistent with previous reports on sulfuric acid‐hydrolyzed cellulose [32], the CNC prepared in our study consists predominantly of short, rigid, rod‐like nanocrystals bearing surface sulfate ester groups (Figure 1C), resulting in high colloidal dispersibility but limited conformational adaptability. Conversely, CNF forms an elongated and entangled fibrillar structure characterized by a high aspect ratio, large specific surface area, and abundant surface hydroxyl groups (Figure 1C). This fibrillar architecture endows CNF with greater structural flexibility, higher viscosity, and enhanced gel‐forming capacity [33], as well as improved morphological stability across the gastrointestinal digestive phases [34, 35]. These properties confer an enhanced interaction potential within the gastrointestinal environment [36]. Previous studies have demonstrated that the extended fibrillar morphology and higher surface hydroxyl group density of CNF facilitate multivalent hydrogen bonding and physical entrapment of bile acids, thereby enhancing bile acid sequestration and limiting their enterohepatic reabsorption [24, 36, 37]. Increased binding and close association with the mucus‐microbiota interface likely underlies the more pronounced CNF‐induced remodeling of microbiota composition and function compared with CNC. Collectively, these considerations suggest that the superior protective effect of CNF relative to CNC may arise from its nanoscale structural attributes, which optimize its interactions with the gastrointestinal tract.

Meanwhile, our findings also demonstrate that nanocellulose intervention profoundly reshapes the gut microbial ecosystem in ICP. The microbiome of ICP rats was predominantly composed of potentially pathogenic bacteria, including *Treponema*, *Eubacterium_ruminantium_group*, and *Eubacterium_xylanophilum_group*, which was associated with gut dysbiosis and disease susceptibility. In contrast, nanocellulose intervention promoted the enrichment of bacterial genera commonly linked to metabolic homeostasis and intestinal health [38, 39]. Notably, CNF induced a distinct microbial profile characterized by increased abundance of *Prevotella*‐ and *Alloprevotella*‐related taxa, consistent with prior reports of nanocellulose‐mediated microbiota modulation in models of intestinal inflammation and constipation [26, 40]. Notably, the fecal microbiota transplantation experiments provided direct causal evidence that gut microbiota remodeling is sufficient to mediate the protective effects of nanocellulose in ICP. Transfer of microbiota from CNF‐treated donors markedly recapitulated the improvement in bile acid burden, liver injury, and pregnancy outcomes observed in donor animals, whereas microbiota from CNC‐treated donors conferred only partial protection. This FMT‐mediated phenotypic transfer mirrors the differential efficacy of CNF and CNC observed in *vivo*, suggesting that the enhanced early prophylactic effect of CNF is mediated by the remodeled gut microbiota.

Emerging evidence underscores the profound impact of gut microbiota on bile acid metabolism within the gut–liver axis [41]. In this study, we observed that most bile acids in the fecal bile acid profiles of the ICP‐CNC and ICP‐CNF groups were significantly elevated compared to the ICP group, suggesting higher levels of bile acids in feces (Figure 4B). Interestingly, the level of LCA in the ICP‐CNF group was notably lower than in the ICP group. Moreover, our findings indicate that the CNF in nanocellulose modulates bile acid metabolism by remodeling the gut microbiota. Compared with the ICP group, CNF intervention was associated with a reduced abundance of bacterial taxa functionally linked to BSH and/or bile acid‐inducible (bai) gene pathways, including *Lactobacillus*, *Clostridium sensu stricto 1*, and *Acetatifactor* [42, 43, 44]. This microbial shift was accompanied by an attenuation of the CDCA‐to‐LCA transforming capacity, thereby favoring CDCA retention and limiting LCA production (Figure S7B) [43, 45]. A possible explanation for this finding is that CNF‐mediated remodeling of the gut microbial community limits its collective 7α‐dehydroxylation capacity. This hypothesis is supported by reports linking the reduced abundance of 7α‐dehydroxylation‐related genes in gut microbiota to decreased intestinal LCA production [46, 47]. Consistent with our finding, fecal CDCA levels were higher in healthy pregnant women than in patients with ICP (Figure 4C). Also consistent with this pattern, CNF appears to reduce the abundance of bacteria responsible for CDCA‐to‐LCA metabolism.

Furthermore, the resulting accumulation of CDCA, a potent endogenous activator of FXR, is likely to promote activation of the intestinal FXR pathway and subsequent induction of its downstream effectors, including SHP and FGF15 [48]. Indeed, we observed a significant upregulation of the expression of *Shp* and *Fgf15* (Figure 7A), at the ileal terminal in the ICP‐CNC and ICP‐CNF groups, indicating induction of intestinal FXR signaling. Likewise, our study revealed that the expression of Asbt, a key transporter involved in bile acid uptake in the ileum, was significantly downregulated in the ICP‐CNF group (Figure 7A and Figure S11), likely due to SHP‐mediated repression [49]. We further measured serum FGF15 levels and found them significantly elevated in the ICP‐CNC and ICP‐CNF groups (Figure 7B), suggesting that intestinal FXR activation promoted FGF15 expression. Meanwhile, FXR antagonism with Z‐guggulsterone markedly attenuated the beneficial effects of CNF on cholestatic phenotypes, supporting a critical role of FXR signaling in CNF‐mediated protection (Figure 6). Thus, these results suggest that nanocellulose administration, particularly CNF, could reduce bile acid, particularly primary bile acid, reabsorption from the terminal ileum into the bloodstream via gut microbiota‐mediated FXR‐dependent bile acid excretion, and promote the release of intestinal FGF15 into the circulation.

Previous studies have suggested that intestinally derived FGF15 can signal to the liver via the portal circulation, bind to the hepatic FGFR4 receptor, and activate the JNK1/2 and ERK1/2 signaling pathways, thereby further repressing bile acid synthesis [50, 51]. Moreover, we observed a significant increase in the expression of hepatic *Fxr*, *Fgf15*, and *Shp* in the ICP‐CNC and ICP‐CNF groups (Figure 7C), indicating activation of the hepatic FXR pathway. The activation of this pathway can also inhibit bile acid synthesis via SHP‐mediated repression of liver receptor homolog‐1 (LRH‐1) [45]. As a result, we found that the expression of CYP7A1, the rate‐limiting enzyme in the classical bile acid synthesis pathway [52], was significantly lower in the ICP‐CNC and ICP‐CNF groups compared to the ICP group (Figure 7C,D). This effect was likely mediated by the activation of the FXR, JNK1/2, and ERK1/2 signaling pathways, further reducing bile acid synthesis. In addition, the activation of FXR signaling in the nanocellulose intervention groups led to a significant upregulation of MRP2 in the ICP‐CNF group and an increase in MRP2 expression in the ICP‐CNC group (Figure 7D), a critical transporter responsible for bile acid excretion into the bile ducts [53], thereby promoting bile acid excretion into the bile canaliculus. Collectively, these findings indicate that nanocellulose intervention modulated the composition and abundance of gut microbiota, activated FXR signaling in the ileum and liver, reduced bile acid reabsorption by downregulating ASBT, suppressed bile acid synthesis by inhibiting hepatic CYP7A1 expression, and promoted bile acid excretion through upregulated MRP2 expression, with CNF demonstrating the most pronounced effects (Figure 7E). Collectively, these findings provide mechanistic insight into how CNF‐mediated microbiota remodeling links altered bile acid biotransformation to restoration of gut‐liver axis homeostasis in ICP.

In addition to significantly influencing bile acid metabolism, gut microbiota play a crucial role in modulating levels of SCFAs, tryptophan metabolites, and TCA cycle‐related intermediates that support gut health [54]. The present study further demonstrates that CNF modulates microbial metabolic functions, thereby extending the functional relevance of nanocellulose from local intestinal homeostasis to systemic metabolic regulation, which distinguishes our findings from previous studies on nanocellulose and conventional dietary fibers. Nanocellulose shares key functional similarities with dietary fibers, particularly in its fermentation‐associated and prebiotic effects on the gut microbiota. Notably, however, these effects are strongly influenced by fiber size and nanoscale architecture. Compared with conventional dietary fibers, nanocellulose with reduced particle dimensions (CNC: ∼114 nm in length and 28 nm in diameter; CNF: ∼340 nm in length and 52 nm in diameter in the present study) exhibits enhanced mucoadhesion, increases luminal viscosity [32, 55], and modulates intestinal transit and nutrient diffusion through gel formation and water‐holding capacity [16, 56, 57]. These physicochemical properties facilitate enhanced cecal fermentation, leading to increased production of microbial metabolites that serve as substrates and signaling molecules for the gut microbiota, which are recognized contributors to metabolic regulation and barrier protection [25]. Our findings show that nanocellulose intervention enhanced gut ecology by increasing SCFAs such as acetate and butyrate, known for their anti‐inflammatory effects [58, 59], as well as propionate and isobutyrate, which contribute to microbial balance [60]. These results are consistent with previous studies showing that nanocellulose supplementation lowers fermentation pH, signifying increased SCFA production [25, 61]. A lower intestinal pH helps protect against pathogenic bacteria and promotes the growth of beneficial gut microbes [38]. The observed SCFA alterations were particularly associated with the elevated abundance of *Prevotella_9*, *Prevotellaceae_UCG‐001*, and *Alloprevotella* in the ICP‐CNF group. Notably, *Prevotella_9* and *Prevotellaceae_UCG‐001* degraded dietary fibers into SCFAs such as acetate, propionate, and butyrate [62], while *Alloprevotella*, a prominent SCFA‐producing bacterium with saccharolytic activity [63], produces additional SCFAs including isobutyrate and isovalerate [64], collectively enhancing gut barrier integrity and reducing inflammation. Furthermore, as a potential prebiotic, nanocellulose promotes probiotic growth, leading to increased SCFA production, which in turn reduces bacterial endotoxins and lowers the risk of liver damage [65]. In addition to modulating SCFA production, nanocellulose intervention, particularly CNF, significantly increased fecal, serum, and hepatic levels of tryptophan metabolites, including gamma‐aminobutyric acid (GABA), quinolinic acid, and indole‐3‐pyruvate. These metabolites play crucial roles in energy metabolism and immune modulation, with GABA exerting anti‐inflammatory effects and strengthening the gut barrier [66]. We also observed increased levels of TCA cycle intermediates, such as citric acid, α‐ketoglutaric acid, succinic acid, and itaconic acid, following nanocellulose intervention indicating enhanced energy metabolism and anti‐inflammatory activity. Notably, itaconate and succinate have been recognized for their immunomodulatory properties [67, 68], suggesting that the benefits of nanocellulose extend beyond metabolic restoration to include inflammation mitigation. These findings highlight the capacity of nanocellulose to reshape gut microbiota, enhance SCFA production, improve tryptophan metabolism, and optimize TCA cycle activity, collectively contributing to gut ecosystem stability.

Importantly, the beneficial effects of nanocellulose observed in the present ICP model need to be considered in the context of its favorable safety profile following both conventional oral and gestational administration. Micron‐scale cellulose fibers or crystals (e.g., microcrystalline cellulose, MCC) have been designated as Generally Recognized as Safe (GRAS) by the U.S. Food and Drug Administration (FDA). Consistent with previous reports demonstrating the biocompatibility and minimal immunogenicity of nanocellulose in *vitro* and in *vivo* [38, 69], our safety assessment revealed no treatment‐related alterations in physiological parameters or histopathological features of major organs following the oral administration of nanocellulose (Figure 1E–H and Figure S1). On the basis of this favorable safety profile, CNF exhibited superior efficacy in ameliorating ICP‐related phenotypes than UDCA, the current first‐line treatment for ICP that acts by reducing bile acid hydrophobicity and cytotoxicity through pharmacological modulation of the host bile acid pool [7]. Notably, the limited efficacy of UDCA in the present model was accompanied by substantial inter‐individual variability, consistent with the heterogeneous therapeutic responses reported in clinical studies [8, 9]. In an additional exploratory experiment initiated after ICP induction, co‐administration of UDCA with CNF did not appear to provide additional benefit compared with CNF alone under the tested conditions (data not shown). Together, these observations indicate that safely administered plant‐derived nanocellulose reshapes the intestinal microenvironment and reprograms microbiota‐driven bile acid transformation, thereby secondarily modulating FXR signaling along the gut‐liver axis and restoring bile acid homeostasis.

Despite the encouraging nature of our findings, several limitations of this study warrant consideration. First, clinical trials are necessary to validate the results, particularly regarding the ability of nanocellulose to regulate bile acid levels and improve perinatal outcomes. Second, although nanocellulose possesses considerable potential as a drug delivery platform, its co‐administration with therapeutic agents was not explored in this study. Third, the present conclusions are based on an EE2‐induced rat model of ICP, and species‐specific differences may limit direct extrapolation to human pregnancy. Fourth, although preliminary dose‐ranging and timing‐of‐intervention analyses were performed, the optimal prophylactic window and human‐equivalent dosing of CNF remain to be defined through systematic pharmacokinetic and translational studies. Fifth, while the microbiota‐FXR axis is mechanistically central, the relative contribution of CNF's physical adsorption capacity remains to be fully quantified. Finally, although maternal and fetal outcomes were comprehensively evaluated, we did not directly quantify nanocellulose biodistribution or organ‐level accumulation (e.g., using ICP‐MS‐based tracer strategies or labeled nanocellulose), nor assess key fetal organ development markers, which should be addressed in future investigations.

## 4 Conclusion

In this study, we investigated the effects of nanocellulose (CNC and CNF) in a rat ICP model and elucidated how nanocellulose‐mediated gut microbiota alterations regulate bile acid metabolism (Figure 7E). By integrating multi‐omics analysis, we demonstrated that nanocellulose, particularly CNF, promotes the expansion of beneficial gut bacteria such as *Prevotella_9* and *Prevotellaceae_UCG‐001*, thereby improving gut ecology. Nanocellulose also suppresses the growth of *Acetatifactor* and *Lactobacillus*, thereby reducing bile acid transformation and promoting the accumulation of CA and CDCA in feces. These microbial shifts activate the FXR signaling pathway along the gut‐liver axis by inhibiting intestinal bile acid reabsorption transporters, downregulating hepatic bile acid synthesis rate‐limiting enzymes, and upregulating bile acid efflux transporters, collectively establishing a feedback regulatory network for bile acid homeostasis. As a result, nanocellulose intervention effectively lowers systemic total bile acid levels and mitigates adverse perinatal outcomes, including improved offspring survival rates. These findings underscore the potential of the diet‐gut microbiota‐bile acid‐FXR axis as a potential target for prophylactic dietary intervention in populations at high risk of ICP.

## 5 Experimental Section

### 5.1 Materials

Twenty‐seven bile acid standards (Table S2) and MCC (microcrystalline cellulose) were purchased from Shanghai Zzbio Co., Ltd. (Shanghai, China). The internal standard (IS), cholic acid‐2,2,4,4‐d (CA‐d4, #20849), was obtained from Cayman Chemical Co. (Ann Arbor, USA). Sulfuric acid and sodium hydroxide were purchased from Chengdu Kelong Chemical Co., Ltd. (Chengdu, China). 17α‐Ethinylestradiol (EE2, #E4876), UDCA (#U5127), methyl chloroformate (MCF, #M35304), and ammonium acetate solution were obtained from Sigma–Aldrich Chemicals (St. Louis, USA). Propylene glycol (#P103433) was sourced from Shanghai Aladdin Biochemical Technology Co., Ltd. (Shanghai, China), and sodium pentobarbital was obtained from Beijing Huaye Huanyu Chemical Co., Ltd. (Beijing, China). LC‐MS grade methanol (MeOH) was obtained from Merck (Darmstadt, Germany), and LC‐MS grade acetonitrile (ACN) was purchased from Thermo Fisher Scientific (Waltham, USA). All aqueous solutions were prepared with deionized water produced by a Milli‐Q Advantage A10 Water Purification System (Merck Millipore, Darmstadt, Germany). Methanol and acetonitrile were of LC‐MS grade, while other chemicals were analytical grade without further purification.

### 5.2 Preparation of Nanocellulose

#### 5.2.1 Preparation of CNC

CNC was prepared from MCC via sulfuric acid hydrolysis [70]. MCC was dispersed in 64% (w/w) sulfuric acid (H_2_SO_4_) at a solid‐to‐liquid ratio of 1:20 (w/v) and hydrolyzed under continuous stirring at 45°C for 1.5 h. The reaction was terminated by diluting the mixture with five times its volume of distilled water. The resulting suspension was centrifuged (5000 rpm, 25°C, 10 min) and repeatedly washed with distilled water until the supernatant appeared turbid. Subsequently, the suspension was dialyzed against distilled water at 4°C for 48 h to remove residual acid, yielding a CNC suspension stored at 4°C for subsequent experiments.

#### 5.2.2 Preparation of CNF

CNF was fabricated from MCC via mechanical ball milling using an NaOH solution as the wet milling medium [71]. Briefly, 1 g of MCC was suspended in 20 mL of 2% NaOH solution and placed in a ball milling jar, followed by the addition of 90 g of zirconia balls (diameters: 15, 12, 10, 8, and 5 mm). The milling process was conducted at 400 rpm and 25°C in an intermittent mode, consisting of 10 min of forward milling, a 10 min pause, and 10 min of reverse milling, repeated for a total milling duration of 2 h. The resulting suspension was dialyzed at 4°C for 48 h to eliminate residual alkali, ultrasonicated to ensure uniform dispersion, and stored at 4°C for subsequent use.

### 5.3 Characterization of Nanocellulose

FTIR spectra of the freeze‐dried CNC and CNF samples were analyzed using an FTIR spectrometer (Nicolet iS10, Thermo Fisher Scientific, USA). Spectral acquisition was conducted with 32 scans at a resolution of 4 cm^−1^ over a wavenumber range of 4000–600 cm^−1^ at 25°C. XRD patterns of the freeze‐dried CNC and CNF were analyzed using an X‐ray diffractometer (X'Pert3 Powder 10300, Bruker, USA) under Cu‐Kα radiation (40 kV, 40 mA) with a scattering angle (2θ) range of 10°–50°, a scanning rate of 2°/min and a step size of 0.02°. The corresponding crystallinity index (CrI) was calculated using the Segal equation [72]. AFM images of CNC and CNF were obtained using an atomic force microscope (NanoMan VS, Bruker, USA). Prior to observation, a drop of a diluted CNC or CNF suspension (0.01%, w/v) was deposited onto freshly mica substrates. After air drying at room temperature, AFM imaging was performed and the average particle dimensions were analyzed using Nano Measure software.

### 5.4 Animal Studies and Experimental Design

#### 5.4.1 Animals, Ethics Statement, and Experimental Design

All animal experiments were conducted in accordance with the Guide for the Care and Use of Laboratory Animals and were approved by the Institutional Animal Care and Use Committee (IACUC) of Chongqing Medical University (Approval No. IACUC‐CQMU‐2023‐0214 and IACUC‐CQMU‐2024‐0438). Specific pathogen‐free female Sprague‐Dawley (SD) rats (8–10 weeks old, 190–230 g) were obtained from the Experimental Animal Center of Chongqing Medical University (Chongqing, China). Animals were housed under controlled conditions (temperature 18°C–21°C, relative humidity 65%–70%, 12 h light/12 h dark cycle) with free access to standard chow (Table S3) and water. All rats were acclimatized for at least one week prior to experimentation. Anesthesia was induced with sodium pentobarbital at a dose of 30 mg/kg.

For pregnancy induction, females were housed with proven fertile males overnight, and the presence of a vaginal plug the following morning was designated as gestational day 1 (GD1). On GD21, fasting blood samples were collected from the fundus venous plexus, centrifuged at 3000 rpm for 10 min at 4°C, and serum was stored at −80°C for biochemical analysis. Cesarean section was subsequently performed to record fetal survival. Portions of maternal or fetal tissues were fixed in 4% paraformaldehyde for histological examination by H&E staining, and the remainder were stored at −80°C for other analyses. Cecal contents were collected aseptically and snap‐frozen in liquid nitrogen immediately and stored at −80°C for microbiome and metabolites profiling.

#### 5.4.2 Long‐Term Safety Assessment of Oral Nanocellulose in Non‐Pregnant Rats

Thirty female SD rats were randomly assigned to three groups (n = 10 per group): Control, CNC, and CNF, for assessment of the systemic safety of orally administered nanocellulose under non‐pregnant conditions. The Control group received deionized water by oral gavage, while the CNC and CNF groups received suspensions of CNC or CNF, respectively, at a dose of 90 mg/kg/day for 8 weeks. Body weight was recorded every two days. At weeks 4 and 8, five rats from each group were fasted overnight. Serum was collected and stored at −80°C for biochemical analyses. Major organs (heart, liver, spleen, lung, kidney, cecum, and colon) were excised and fixed in 4% paraformaldehyde.

#### 5.4.3 Dose‐ and Timing‐Optimization of CNF Intervention in the ICP Rat Model

Experimental ICP was induced using 17α‐ethinylestradiol (EE2), a well‐established rodent model [73, 74, 75, 76]. EE2 was dissolved in propylene glycol at a final concentration of 1.0 mg/mL. Pregnant rats received subcutaneous injections of EE2 (5 mg/kg/day) from GD13 to GD20 to induce and maintain cholestasis, while control pregnant rats received vehicle alone.

To determine the optimal effective dose and the most appropriate intervention window of CNF, a combined dose‐response and timing‐of‐administration study was performed. For the dose‐response experiment, twenty‐five female Sprague‐Dawley rats (9–10 weeks old) were randomly assigned to five groups (n = 5 per group): Control, ICP, and three CNF‐treated groups receiving 60, 90, or 120 mg/kg/day, respectively. From GD1 onward, rats in the three CNF groups received daily oral gavage of CNF at the indicated doses, while the Control and ICP groups were administered an equal volume of deionized water. Except for the Control group, which received propylene glycol vehicle only, all other groups were subjected to ICP induction by subcutaneous injection of EE2 (5 mg/kg/day) from GD13 to GD20.

To evaluate the influence of intervention timing, an additional cohort of twenty‐five female Sprague‐Dawley rats (9–10 weeks old) was randomly allocated into five groups (n = 5 per group): Control, ICP, Pre‐Gestational Day 14 (Pre‐GD14), GD1, and GD13. Rats in the Pre‐GD14 group received oral gavage of CNF (90 mg/kg/day) starting 14 days prior to mating and continuing throughout gestation. Rats in the GD1 and GD13 groups received the same dose of CNF beginning on GD1 and GD13, respectively, whereas the Control and ICP groups were given equal volumes of deionized water. With the exception of the Control group, which received propylene glycol vehicle only, all other groups were subjected to ICP induction by subcutaneous injection of EE2 (5 mg/kg/day) from GD13 to GD20.

#### 5.4.4 Nanocellulose Intervention in the EE2‐Induced ICP Rat Model

Based on preliminary dose‐response and timing experiments in the EE2‐induced ICP rat model, CNF administration at 90 mg/kg/day starting 14 days prior to pregnancy produced the most robust reduction in serum bile acid levels. This dosing regimen was therefore selected for subsequent experiments unless otherwise specified (Figure S2). Moreover, according to published preclinical evidence and with consideration of drug safety during pregnancy, UDCA was administered at a dose of 25 mg/kg/day in this study [73, 74].

For the main intervention study, thirty pregnant rats were randomly allocated to five groups (n = 6 per group): Normal pregnancy (NP), ICP, ICP+UDCA, ICP+CNC, ICP+CNF. CNC and CNF were administered by oral gavage at 90 mg/kg/day starting 14 days before mating and continued throughout gestation unless otherwise specified. The ICP+UDCA group received UDCA at 25 mg/kg/day by oral gavage from GD17 to GD20. Body weight was monitored every two days. On GD21, after overnight fasting, rats were anesthetized with pentobarbital (30 mg/kg).

#### 5.4.5 FMT in Pregnant ICP Rats

Pregnant rats received FMT from CNC‐ or CNF‐treated donors following a previously reported protocol [77]. Twenty female Sprague‐Dawley rats (9–10 weeks old) were randomly assigned to four groups (n = 5 per group): Control, ICP, ICP+CNC^FMT^, and ICP+CNF^FMT^. To deplete endogenous gut microbiota, rats in the ICP+CNC^FMT^ and ICP+CNF^FMT^ groups received an antibiotic cocktail consisting of vancomycin (100 mg/kg), neomycin sulfate (200 mg/kg), metronidazole (200 mg/kg), and ampicillin (200 mg/kg) by oral gavage once daily from GD3 to GD6. Fresh fecal samples were collected from donor rats in the ICP‐CNC and ICP‐CNF groups and initially suspended in sterile PBS at 200 mg/mL, filtered through a 200 µm mesh to remove particulate matter, and then subjected to a low‐speed centrifugation step at 800 × g for 10 min to sediment large debris. The supernatant was centrifuged at 6000 × g for 20 min at 4°C to collect microbial pellets, followed by three washes with sterile PBS and resuspension in sterile PBS to a fecal‐equivalent concentration of 800 mg/mL. The bacterial suspension was then mixed with an equal volume of sterile 20% glycerol solution, yielding a final fecal suspension concentration of 400 mg/mL and a final glycerol concentration of 10% (v/v), and stored at −80°C until use. Additionally, AFM test was used to confirm the absence of residual nanocellulose in the bacterial suspensions (Figure S13), with a lateral resolution of ∼0.1–0.2 nm under our imaging conditions. From GD7 to GD20, recipient rats in the ICP+CNC^FMT^ and ICP+CNF^FMT^ groups were orally gavaged once daily with 2 mL of freshly thawed fecal suspension (400 mg/mL). Control and ICP groups received an equal volume of sterile deionized water. To induce ICP, rats in the ICP, ICP+CNC^FMT^, and ICP+CNF^FMT^ groups received subcutaneous injections of EE2 (5 mg/kg/day) from GD13 to GD20, while Control rats received propylene glycol vehicle alone.

#### 5.4.6 FXR Antagonist Intervention With Z‐Guggulsterone

An FXR antagonist intervention was performed using Z‐guggulsterone (Cat# HY‐110066, MCE) to inhibit FXR signaling in CNF‐treated ICP rats. Twenty female Sprague‐Dawley rats (9 weeks old) were randomly allocated into four groups (n = 5 per group): Control, ICP, ICP+CNF, and ICP+CNF+Z‐guggulsterone. After one week of acclimatization, rats in the ICP+CNF and ICP+CNF+Z‐guggulsterone groups received daily oral gavage of CNF suspension (90 mg/kg/day). In addition, rats in the ICP+CNF+Z‐guggulsterone group were co‐treated with the FXR antagonist Z‐guggulsterone at a dose of 10 mg/kg/day by oral gavage, whereas the Control and ICP groups received an equal volume of deionized water. From GD13 to GD20, ICP was induced in the ICP, ICP+CNF, and ICP+CNF+Z‐guggulsterone groups by subcutaneous injection of EE2 (5 mg/kg/day), while the Control group received propylene glycol vehicle.

#### 5.4.7 Maternal and Fetal Safety Assessment During Gestation

An additional full‐gestation exposure study was conducted in pregnant rats to examine maternal and fetal safety following oral nanocellulose administration. Nine female Sprague‐Dawley rats (10 weeks old) were randomly assigned to three groups (n = 3 per group): Control, CNC, and CNF. Pregnant rats in the CNC and CNF groups received daily oral gavage of suspensions of CNC or CNF, respectively, at a dose of 90 mg/kg/day throughout gestation, whereas Control rats were administered an equal volume of deionized water.

#### 5.4.8 Expanded‐Dose UDCA Intervention in the ICP Rat Model

An additional cohort of thirty female Sprague‐Dawley rats (9 weeks old) was randomly assigned to five groups (n = 6 per group): normal pregnancy (NP), ICP, ICP treated with UDCA at 25 mg/kg/day (ICP+UDCA_25), ICP treated with UDCA at 50 mg/kg/day (ICP+UDCA_50), and ICP treated with CNF (ICP+CNF). The NP and ICP groups were established as described above. In the ICP+UDCA_25 and ICP+UDCA_50 groups, UDCA was administered by oral gavage at 25 or 50 mg/kg/day, respectively, from GD17 to GD20 following ICP induction. In the ICP+CNF group, CNF was administered by oral gavage at 90 mg/kg/day starting 14 days before mating and continued throughout gestation.

### 5.5 Study Population and Sample Collection

Participants were recruited from the Second Affiliated Hospital of Chongqing Medical University, China between March 2023 and April 2024. The primary inclusion criteria for ICP were fasting serum TBA levels ≥10 µmol/L and unexplained pruritus. The exclusion criteria were as follows: (1) confirmed viral infections, the presence of hepatitis virus or Epstein‐Barr virus; (2) underlying liver or gallbladder diseases confirmed by ultrasound; (3) gestational diabetes mellitus, multiple pregnancies, or significant thyroid dysfunction; and (4) current use of oral UDCA therapy. Ethical approval was granted by the Ethics Committee of the Second Affiliated Hospital of Chongqing Medical University (Approval No.202483), and written informed consent was obtained from all participants prior to enrollment. Blood samples were collected following an overnight fast of at least 8 h, centrifuged at 3000 rpm for 10 min at 4°C to isolate serum, and stored at −80°C until analysis. Participants were also provided with a sterile disposable bedpan and fecal collection tube in advance. Standardized instructions for fecal sample collection were given by a trained clinician to ensure procedural consistency. Participants were instructed to collect the sample within 10 min of defecation, immediately seal the tube, and place it into a pre‐chilled insulated container with ice packs. All samples were delivered to the laboratory under cold‐chain conditions within 4 h of collection and were promptly stored at −80°C until further processing.

### 5.6 Histopathological Assessment

Tissue sections (lung, heart, liver, spleen, kidney, caecum, and colon) were fixed in 4% paraformaldehyde for 24 h at 4°C, followed by dehydration through a series of ascending concentrations of ethyl alcohol and dimethylbenzene. After embedding in paraffin, samples were sectioned at a thickness of 4 µm. These sections were floated on 40°C water using a model KD‐P spreading machine (Kedee Instruments Co., Ltd, Zhejiang, China), collected on glass slides, and then heated in an oven at 60°C until water was evaporated and paraffin melted. The dewaxing process was performed using dimethylbenzene and ethyl alcohol, followed by staining with hematoxylin and eosin. Subsequently, sections were dehydrated in ethyl alcohol and dimethylbenzene again and finally sealed with neutral gum. Histopathological analysis was conducted using a Leica DM6 B upright digital microscope and LAS X 4.7.0 software (Leica Microsystems GmbH, Wetzlar, Germany).

### 5.7 Immunofluorescence Imaging of Tissue Sections

Paraffin‐embedded intestinal sections were deparaffinized using a dewaxing reagent (10 min × 3 cycles), followed by graded ethanol dehydration and rinsing with distilled water. Antigen retrieval was performed using EDTA buffer (pH 8.0) with microwave heating (medium power for 8 min, resting for 8 min, followed by low‐medium power for 7 min). After cooling, the sections were washed with phosphate‐buffered saline (PBS, pH 7.4). Tissue regions were delineated using a hydrophobic barrier pen, followed by blocking with 5% bovine serum albumin (BSA) at room temperature for 30 min. Subsequently, the sections were incubated overnight at 4°C in the dark with a primary antibody against apical sodium‐dependent bile acid transporter (ASBT) (rabbit anti‐ASBT, 1:4000, #DF12351, Affinity Biosciences LTD, Cincinnati, USA). The next day, after PBS washing, a goat anti‐rabbit secondary antibody (1:100) was applied, followed by incubation at room temperature for 50 min. Nuclei were counterstained with 4',6‐diamidino‐2‐phenylindole (DAPI) in the dark for 10 min. Autofluorescence was quenched using a dedicated quenching reagent (5 min), followed by thorough rinsing under running water for 10 min. Finally, the sections were washed with PBS, mounted with an anti‐fade mounting medium, and examined using a Nikon upright fluorescence microscope (Tokyo, Japan). Nuclei were stained blue with DAPI, and target proteins showing positive signals were visualized in red.

### 5.8 16S rDNA Sequencing of Gut Microbiota

Cecum contents from rats were collected and immediately frozen at −80°C. Fecal bacterial DNA was extracted using a TIANamp Soil DNA Kit (Cat#DP336, Tiangen Biotech Co. Ltd., China), following the manufacturer's instructions. The quantity and quality of the DNA were assessed using a Nucleic Acid Fragment Analyzer System (Agilent 5400). The V3‐V4 regions of the 16S rRNA gene were amplified using the universal primers 341F (5′‐CCTAYGGGRBGCASCAG‐3′) and 806R (5′‐GGACTACNNGGGTATCTAAT‐3′). All PCRs were performed with 15 µL of Phusion High‐Fidelity PCR Master Mix (Biolabs, USA), 0.2 µmol/L of forward and reverse primers, and approximately 10 ng of template DNA. The thermal cycling protocol consisted of an initial denaturation step at 98°C for 1 min, followed by 30 cycles of denaturation step (98°C for 10 s), annealing step (50°C for 30 s), elongation step (72°C for 30 s), and a final extension step at 72°C for 5 min. The PCR products were then purified using magnetic bead purification. Sequencing libraries were established using the NEBNext Ultra II DNA Library Prep Kit (Cat#E7645B, Biolabs, USA) following the manufacturer's instructions and were sequenced on the NovaSeq6000 PE250 platform by Novogene (Beijing, China).

### 5.9 Bile Acids Profiling by Liquid Chromatographic‐Tandem Mass Spectrometry (LC‐MS/MS)

#### 5.9.1 Preparation of Stocks, Working Solutions, and Calibration Curves

Bile acid (BA) standard stock solutions and internal standard (IS) stock solutions were prepared by dissolving the respective 27 BAs and CA‐d4 in methanol to obtain individual stock solutions of 1 mg/mL and stored at −80°C. The individual BA stock solutions were mixed and diluted in methanol to create a working solution containing each BA at 10 ppm. Furthermore, a mixture of the IS working solution, prepared by using ice‐cold ACN:deionized water (1:1 v/v), was added to both biological samples and the calibration curve, resulting in a final concentration of 50 ppb. Subsequently, nine‐point calibration curves (5, 10, 20, 50, 100, 200, 500, 800, and 1000 ppb) were prepared to quantify BAs.

#### 5.9.2 Sample Preparation

Serum samples (100 µL) were extracted using 500 µL of ACN/MeOH (1:1 v/v) supplemented with CA‐d4. Extraction was performed with continuous shaking for 1 min at 2000 rpm in a cold metal block using a Vortex3000 (Wiggens, Germany), followed by centrifugation at 13 000 rpm at 4°C for 10 min. Finally, 200 µL of the supernatants were transferred to a 250 µL polypropylene vial prior to mass spectrometry analysis.

Liver samples (100 ± 5 mg) were homogenized using three tungsten carbide beads (3 mm diameter) on the TissueLyser II (Qiagen, USA) with three cycles (30 s at 30 Hz, 30 s break between each cycle) in 900 µL of ACN. The homogenates were centrifuged at 13 000 rpm at 4°C for 10 min. 300 µL of the supernatant was placed in a 2 mL amber vial (Agilent, USA) with 270 µL of deionized water and 30 µL of the internal standard mixture.

Fecal samples (100 ± 5 mg) were extracted using 900 µL of ACN supplemented with CA‐d4. Extraction was performed with continuous shaking for 2 min at 2000 rpm and then centrifuged at 13 000 rpm at 4°C for 10 min. A portion (25 µL) of the fecal supernatant was placed in a 2 mL amber vial (Agilent, USA), followed by the addition of 925 µL of ACN/water (1:1 v/v) and 50 µL of the internal standard mixture. Subsequently, 300 µL of the fecal supernatant was placed in a 2 mL amber vial (Agilent, USA), along with 270 µL of deionized water and 30 µL of the internal standard mixture.

#### 5.9.3 LC‐MS/MS Parameters and Analytical Conditions

LC‐MS/MS analysis was conducted using an Agilent 1290 UPLC coupled with an Agilent 6495 triple quadrupole mass spectrometer equipped with an electrospray ionization (ESI) source (Agilent Technologies, USA). Nitrogen was used as both the drying gas and the nebulizing gas, with desolvation gas flow at 15 L/min, sheath gas at 12 L/min, and a nebulizing pressure of 45 psi. The desolvation temperature was maintained at 290°C, and sheath gas temperature at 400°C. The capillary voltage was optimized for each segment from 4000 to 4500 V. The nozzle voltage was set at 1500 V and cell accelerator voltage at 2 V. Chromatographic separation of bile acids was achieved using a reverse‐phase column (RRHD Eclipse Plus 95Å C18, 3 × 150 mm, 1.8 µm, Agilent Technologies, USA) at a flow rate of 0.45 mL/min, 5 µL injection volume, and a column temperature of 40°C. The mobile phase consisted of deionized water with 10 mm ammonium acetate (solvent A) and ACN in a 1:1 ratio (solvent B). Separation was conducted using a gradient, followed by an additional 3‐min post‐run phase to re‐equilibrate the column to initial conditions. Data acquisition used the dMRM mode. At least two transitions (quantifier and qualifier transitions) were selected for each compound in negative ESI mode, depending on the compound. Collision energy was individually optimized for each transition.

### 5.10 Assessment of BSH Activity and 7α‐Dehydroxylation

Fecal samples were preprocessed according to previously reported procedures [78]. Briefly, approximately 100 mg of fecal samples separately collected from ICP, ICP‐CNC, and ICP‐CNF donor rats were suspended in 2 mL of PBS supplemented with 20 mm 2‐mercaptoethanol (ME) to maintain enzymatic activity under reducing conditions, followed by incubation for 30 min at 37°C and mechanical disruption by vortexing. For assessment of BSH activity, a portion of the suspension was filtered through a 200 µm mesh and centrifuged at 6000 × *g* for 15 min at 4°C. The supernatant was collected and analyzed using a commercial BSH activity assay kit (Cat# MK0015RA, MEIKEbio) according to the manufacturer's instructions.

To assess the microbial capacity for 7α‐dehydroxylation‐mediated bile acid transformation, another portion of the suspension was processed following a previously described protocol [79]. The suspension was filtered through a 200 µm mesh and centrifuged at 12 000 × g for 10 min at 4°C. The supernatant was discarded, and the bacterial pellet was washed twice with PBS. Part of the washed pellet (2 mg) was resuspended in 2 mL of 0.1 m PBS containing 500 µg of CDCA and incubated under strictly anaerobic conditions at 37°C for 48 h. The reaction was terminated by adding 200 µL of 15% (w/v) trichloroacetic acid on ice. All sample processing steps were performed under strictly anaerobic conditions. After centrifugation at 16 000 × *g* for 5 min at 4°C, the supernatant was collected and subjected to LC‐MS/MS analysis for quantification of bile acid metabolites.

### 5.11 Metabolomic Profiling by GC‐MS

#### 5.11.1 Sample Preparation and MCF Derivatization

Briefly, 150 µL serum and 20 mg of solid samples (fecal, liver, and gut) were thawed on ice at 4°C. To each sample was added 0.4 mL of a 1 m sodium hydroxide and methanol (1:1 v/v) mixture, along with 10 µL of D4‐alanine (10 mm) and three tungsten carbide beads (3 mm diameter) for solid samples, followed by vortex mixing for 30 s. Solid samples underwent further homogenization using a TissueLyser II (Qiagen, USA) at 30 Hz for 1 min. The supernatant was then isolated by centrifugation at 12 000 rpm for 15 min at 4°C, and stored at 4°C prior to derivatization. Initially, 34 µL of pyridine and 20 µL MCF were successively added to the isolated supernatant, each followed by 30 s of vortex mixing. Subsequently, 400 µL of chloroform and 400 µL of sodium bicarbonate (50 mm) were added and the solution vortexed for 10 s. After centrifugation at 2000 rpm for 10 min, the aqueous layer was discarded, and the remaining chloroform extract was dehydrated by adding approximately 0.3 g of sodium sulfate, then transferred to an amber glass GC‐MS vial. Negative controls were prepared by subjecting a blank microcentrifuge tube to identical processing steps as the samples.

#### 5.11.2 GC‐MS Parameters and Analytical Conditions

GC‐MS was undertaken as previously described [80]. Derivatized metabolites were analyzed using an Agilent Intuvo 9000 gas chromatograph coupled to an MSD 5977B detector with 70 eV electron impact ionization. A DB‐1701 capillary column (20 m × 180 µm id × 0.18 µm, Agilent) was used. Derivatized metabolites were injected into a pulsed splitless mode inlet at 300°C with a helium flow rate of 1 mL/min. The GC oven program started at 45°C for 2 min, then ramped at 9°C/min to 180°C, held for 5 min, ramped at 40°C/min to 220°C, held for 5 min, ramped at 40°C/min to 240°C, held for 11.5 min, and finally ramped to 280°C at 80°C/min. Temperatures for the guard chip, auxiliary, MS quadrupole, and MS source were 300°C, 250°C, 230°C, and 150°C, respectively. Mass detection ranged from 30–550 µm, with a scan speed of 1.563 µ/s and a solvent delay until 4.5 min.

### 5.12 SCFA Quantification in Cecal Samples by Solid Phase Micro‐extraction (SPME)‐GC‐MS

#### 5.12.1 Preparation of Working Solutions and Sample Preparation

The SPME method for SCFA analysis was adapted from Fiorini et al. (2020) [81]. The efficiency and reproducibility of SCFA extraction were enhanced by using a salt solution containing (NH_4_)_2_SO_4_/NaH_2_PO_4_ in a 3.7:1 ratio (1.26 g/mL) and the internal standard D4‐acetic acid (0.5 mm). Fecal samples (20 mg) were placed in a 2 mL screw cap tube containing 400 µL of the salt solution and 3 mm diameter tungsten carbide beads. Homogenization was performed using a TissueLyser II (Qiagen, USA), followed by transfer into a 20 mL glass vial containing 1.6 mL of the salt solution. The sample vials were then placed in the PAL RTC autosampler (Agilent, USA) of the SPME‐GC‐MS instrument for subsequent analysis.

#### 5.12.2 SPME‐GC‐MS Configuration and Parameter Settings

SCFAs were extracted by SPME using a DVB/CAR/PDMS fiber (Agilent) operated on an Agilent PAL RTC 120 autosampler, separated on a 5977A MSD GC (Agilent) equipped with a DB‐FFAP column (30 m × 250 µm i.d. × 0.25 µm, Agilent), and analyzed on a 7890B mass spectrometer (Agilent). SPME conditions were as follows: agitation temperature of 35°C with an extraction time of 30 min. Temperature and desorption time were set at 260°C and 5 min, respectively. Post‐injection fiber conditions were maintained at 270°C for 10 min.

GC‐MS conditions were as follows: Volatile compounds were injected in splitless mode at 260°C with a 1 mL/min helium flow rate. The GC oven program started at 35°C for 4 min, ramped up to 130°C at 70°C/min, increased to 155°C at 5°C/min, and finally to 240°C at 120°C/min, holding for 4 min, resulting in a total run time of 15.06 min. Auxiliary, MS quadrupole, and MS source temperatures were set at 250°C, 230°C, and 150°C, respectively. Mass detection ranged from 30–550 µm, with a scan speed of 1.563 µ/s and a solvent delay until 5.0 min.

### 5.13 MS Data Extraction and Quality Control

#### 5.13.1 GC‐Ms

Total ion chromatograms for SPME and GC‐MS samples were automatically processed using the Automated Mass Spectral Deconvolution & Identification System (AMDIS) software. Target metabolites were identified based on their retention times and mass spectral peaks, matched against an in‐house MS library constructed with authentic chemical standards. The intensity of each chromatographic peak was extracted based on the integrated area of the most abundant reference ion, and subsequently processed using MassOmics‐R, an in‐house R‐based bioinformatics tool. Absolute concentrations of target metabolites in each sample were determined using calibration curves generated from authentic chemical standards, with stable isotope‐labeled internal standards employed for signal normalization and matrix effect correction.

#### 5.13.2 LC‐QqQ

Mass spectrometric data were acquired using Agilent MassHunter Data Acquisition software in dynamic multiple reaction monitoring (dMRM) mode. This mode enabled optimized monitoring of target transitions within expected retention time windows, improving sensitivity and data quality. Qualitative identification of bile acids was based on matching both retention times and MRM transitions to an in‐house bile acid library. Quantitative analysis was conducted using Agilent MassHunter Quantitative Analysis software, applying external calibration curves and stable isotope‐labeled internal standards for matrix effect correction and signal normalization.

#### 5.13.3 Quality Control

Blank samples were analyzed to identify and eliminate background noise, contamination, and residual effects, ensuring data reliability. Concentration normalization was performed in three steps: initial normalization was conducted using the concentration of internal standards, followed by weight‐based normalization to further improve quantification accuracy. Finally, to minimize batch‐specific variations, three quality control (QC) samples were included per batch, and their median values were used for correction. This three‐step approach enhances quantitative robustness and mitigates potential biases introduced by instrumental fluctuations or human variability.

### 5.14 Real‐Time Quantitative PCR (RT‐qPCR) Analysis

Total RNA was extracted from rat tissues and cultured cells using TRIzol reagent (Accurate Biotechnology, Changsha, China) according to the manufacturer's instructions. RNA purity and concentration were determined with a NanoDrop 2000 spectrophotometer (Thermo Fisher Scientific, Waltham, MA, USA). Complementary DNA (cDNA) was synthesized using PrimeScript RT Master Mix (Takara, Dalian, China). Quantitative PCR was performed on a CFX96 Real‐Time PCR Detection System (Bio‐Rad, Hercules, CA, USA) using TB Green Premix Ex Taq II (Takara, Dalian, China). Each sample was analyzed in triplicate. Relative gene expression levels were calculated using the comparative threshold cycle (ΔCt) method, with β‐actin or GAPDH used as internal reference genes. Primer sequences are provided in Tables S4 and S5.

### 5.15 Western Blotting Analysis

Total protein was extracted from rat tissues and cultured cells. Samples were homogenized in RIPA lysis buffer (Beyotime Biotechnology, Jiangsu, China) supplemented with phosphatase and protease inhibitor cocktails (Thermo Fisher Scientific, Waltham, MA, USA) and incubated on ice for 30 min. Lysates were centrifuged at 12 000 rpm for 15 min at 4°C, and the supernatants were collected. Protein concentrations were determined using a BCA assay kit. Equal amounts of protein were separated by SDS‐PAGE (7% resolving gels for proteins >150 kDa and 10% gels for proteins between 30 and 150 kDa) and transferred onto PVDF membranes. After blocking with 5% nonfat milk in TBST (Tris‐buffered saline containing 0.1% Tween‐20), membranes were incubated overnight at 4°C with primary antibodies against CYP7A1 (Affinity Biosciences, #DF2612), CYP27A1 (Abcam, #ab126785), MRP2 (Biodragon Inc., #BD‐PT2840), and ASBT (Huabio, #ER1903‐97). Membranes were then incubated with horseradish peroxidase (HRP)‐conjugated secondary antibodies (1:5000) for 2 h at room temperature. Protein bands were visualized using enhanced chemiluminescence (ECL) reagents. GAPDH or tubulin (Proteintech) served as loading controls. Band intensities were quantified using ImageJ software.

### 5.16 Analysis of Serum Biomarkers

Serum levels of TBA, ALT, AST, BUN, sUA, sCR, GLUT, TC, HDL, and LDL were quantified using a Beckman Coulter AU5821 chemistry analyzer (Shizuoka, Japan).

### 5.17 Measurement of FGF15/19 in Serum or Cell Culture Supernatants

Serum FGF15 (rat) and FGF19 (human) concentrations were determined using commercially available enzyme‐linked immunosorbent assay (ELISA) kits (Rat FGF15: #JM‐5595H1; Human FGF19: #JM‐0787H1; JingMei Biotechnology Co., Ltd., Jiangsu, China) according to the manufacturers’ protocols. Briefly, standard solutions were serially diluted to generate calibration curves, and blank, standard, and sample wells were prepared in parallel. For standard wells, 50 µL of each standard solution was added, whereas sample wells received 40 µL of sample dilution buffer and 10 µL of serum or conditioned medium. Plates were sealed and incubated at 37°C for 30 min, followed by five washes with 30× diluted wash buffer. Subsequently, 50 µL of horseradish peroxidase (HRP)‐conjugated detection antibody was added to each well and incubated under the same conditions, followed by another washing step. Then, 50 µL of tetramethylbenzidine (TMB) substrate solution was added and incubated in the dark for 10 min to allow color development. The reaction was terminated by the addition of the stop solution, and absorbance was measured at 450 nm using a microplate reader. FGF15/19 concentrations were calculated from the corresponding standard curves, with quality control samples included in each assay to ensure accuracy and reproducibility.

### 5.18 In *Vitro* Validation of the Intestinal FXR‐FGF19‐Hepatic FXR Axis

To validate the proposed gut–liver FXR signaling cascade at the cellular level, in *vitro* experiments were performed using human intestinal and hepatic cell models, as previously described [82, 83] with minor modifications. The human colon epithelial cell line Caco‐2 was cultured in DMEM supplemented with 20% fetal bovine serum (FBS) at 37°C in a humidified atmosphere containing 5% CO_2_, and treated with CDCA (100 µm) or the selective FXR agonist GW4064 (1 µm; Cat. No. HY‐50108, MCE) for 24 h. Total RNA and protein were extracted for qPCR and Western blot analyses of FXR signaling‐related genes and proteins.

To examine FXR‐dependent endocrine communication from the intestine to the liver, conditioned media collected from CDCA‐treated Caco‐2 cells was mixed 1:1 with fresh hepatocyte culture medium and applied to primary human hepatocytes for 24 h, with vehicle‐conditioned media serving as the negative control. Hepatocytes treated directly with GW4064 (1 µm) were used as a positive control for hepatic FXR activation. After incubation, hepatocytes were harvested for qPCR and Western blot analyses of FXR target genes and proteins involved in bile acid synthesis and transport.

### 5.19 Statistical Analysis

Data are presented as mean ± SEM. Statistical analysis was performed using Microsoft Excel (2022) and GraphPad Prism (Version 9.5, GraphPad Software Inc., San Diego, USA). *p*‐values were determined using one‐way ANOVA, followed by Tukey's post hoc test or Games–Howell multiple comparisons. *p* < 0.05 was considered statistically significant (*p < 0.05, **p < 0.01, ***p < 0.001).

Sequence data analyses for microbiome results were conducted using QIIME2 and R packages (v4.0.3). Alpha diversity, including observed features, Shannon index, and Simpson index, were analyzed and visualized using the Novogene website (https://magic.novogene.com). Beta diversity analysis was visualized through principal coordinate analysis (PCoA). Linear discriminant analysis effect size (LEfSe) was performed to identify differentially abundant taxa across groups, and the linear discriminant analysis (LDA) score was calculated for taxa that were differentially abundant between groups. A taxon with *p* < 0.05 (Kruskal–Wallis test) and an LDA score greater than 4 was considered significant.

Target metabolite levels were adjusted using log transformation and Pareto scaling to achieve the best Gaussian distribution before statistical analysis. False discovery rates (FDRs) were calculated using the *q‐*value function in R to account for multiple comparisons. Partial least squares discriminant analysis (PLS‐DA) was conducted using the MetaboAnalyst 5.0 package for R (http://www.metaboanalyst.ca) to identify significant metabolites and metabolic profile differences between groups (statistical significance was set at *p* < 0.05 and FDR < 0.3). Similar metabolites between groups were visualized using a petal plot and Venn diagram (OmicStudio cloud platform, https://www.omicstudio.cn), while differential metabolites were visualized using a volcano plot (CNSknowall cloud platform, https://cnsknowall.com). Heatmaps and bubble plots were generated using the ggplot2 and GOplot R packages [84, 85]. Metabolite pathway activity was calculated using the KEGG database with MetaboAnalyst 5.0. The Sankey diagram was used to visualize the relationships between tissue samples, key differential metabolites, and metabolic pathways using Origin software (Version 2025, OriginLab Corporation, MA, USA). Spearman's correlation analysis in R was used to assess the relationship between gut microbiota and clinical characteristics or metabolites.

## Author Contributions

M.Y., H.D., and T.L.H. were responsible for the conceptualization of the study. M.Y., H.D., X.Z., Q.L., H.Y., and T.L.H. contributed to the methodology. H.D., Y.Z., X.D., and T.L.H. acquired funding for the project. T.L.H. managed the project administration. M.Y. and H.D. led the investigation. Y.Z., X.D., and T.L.H. provided resources for the study. M.Y., H.D., Y.Z., X.D., and T.L.H. were involved in data curation. M.Y., Y.Y., D.H., L.Z., R.R., T. He, Y.H., S.V.B., and S.T. contributed to the validation of the results. M.Y., H.D., and H.Y. performed the formal analysis and visualization of the data. M.Y. and H.D. wrote the original draft of the manuscript, while M.Y., H.D., R.D.C., B.N., R.S., and T.L.H. participated in writing, reviewing, and editing the manuscript. Y.Z., X.D., and T.L.H. supervised the study.

## Conflicts of Interest

The authors declare no conflicts of interest.

## Supporting information




**Supporting File**: advs75971‐sup‐0001‐SuppMat.docx.

## Data Availability

The data that support the findings of this study are available from the corresponding author upon reasonable request.
