# Supplementary material for: Nanocellulose Alleviates Intrahepatic Cholestasis of Pregnancy via Gut Microbiota‐Mediated Bile Acid Homeostasis
Source: Adv Sci (Weinh). 2026 Jun 9;13(45):e18337. doi: 10.1002/advs.202518337 (PMC13336375; doi:10.1002/advs.202518337)
Supplement: Supplementary file 1 [file ADVS-13-e18337-s001.docx]

Supporting Information

**Nanocellulose Alleviates Intrahepatic Cholestasis of Pregnancy via Gut Microbiota-Mediated Bile Acid Homeostasis**

*Muhua Yu, Hongjie Dai, Xiaocui Zhong, Qibin Li, Hui Yuan, Yang Yang, Daiyong Huang, Lei Zhang, Rui Ran, Tian He, Yuanzhi Huang, Silas Villas-Boas, Sergey Tumanov, Richard D. Cannon, Boris Novakovic, Richard Saffery, Yuhao Zhang,* Xiaojing Dong,* and Ting-Li Han**

**Supplementary Figures**

**Figure S1. Maternal-fetal safety of oral administration of nanocellulose during pregnancy.** (A-E) Maternal serum biochemical parameters, including markers of liver and kidney function, lipid metabolism, and cardiac injury after continuous nanocellulose administration during gestation. (F) Maternal organ weights, fetal body weights, and placental weights. (G) Representative H&E-stained sections of major maternal tissues. (H) Representative H&E-stained sections of major fetal tissues. Data in (A-F) are presented as mean ± SEM (n = 3 per group). Statistical significance was determined by one-way ANOVA followed by Tukey’s multiple-comparisons test. Scale bar, 100 μm. *P < 0.05, **P < 0.01, ***P < 0.001.

**Figure S2. Dose- and timing-dependent effects of CNF intervention in the ICP rat model.** (A) Serum TBA, AST, and ALT levels following different doses of CNF. (B) Serum TBA, AST, and ALT levels following CNF administration initiated at Pre-Gestational Day 14 (Pre-GD14), Gestational Day 1 (GD1), and Gestational Day 13 (GD13). Data are presented as mean ± SEM. Statistical significance was determined by one-way ANOVA followed by Tukey’s multiple-comparisons test. *P < 0.05, **P < 0.01, ***P < 0.001, ****P < 0.0001.

**Figure S3. Dose-escalation of UDCA compared with CNF treatment in the ICP rat model.** (A) Serum TBA, AST, and ALT and (B) live birth rate in NP, ICP, ICP+UDCA (25 mg/kg/day), ICP+UDCA (50 mg/kg/day), and ICP+CNF groups. Data are presented as mean ± SEM. One-way ANOVA with Tukey’s multiple-comparisons test. *P < 0.05, **P < 0.01, ***P < 0.001, ****P < 0.0001.

**Figure S4. Effects of preconceptional nanocellulose intervention on placental histology and fetal body weight in ICP rats.** (A) Representative H&E-stained placental sections. (B) Fetal body weight. Data in (B) are presented as mean ± SEM. Statistical significance was determined using Welch’s ANOVA followed by Games-Howell’s multiple-comparisons test. Scale bar, 100 μm. *P < 0.05, **P < 0.01.

**Figure** **S5. Differences in gut microbial genera among groups.** Significantly different genera between the ICP group and the NP group (**A**), the ICP–UDCA group (**B**), and the ICP–CNC group (**C**), respectively.

**Figure S6. Effects of nanocellulose on bile acid profiles in liver tissues and serum.** (**A**) Composition of bile acids in liver samples. (**B**) Concentrations of individual bile acid species in liver samples. (**C**) Composition of bile acids in serum samples. (**D**) Concentrations of individual bile acid species in serum samples. Data are presented as mean ± SEM. P values were determined using one-way ANOVA with Tukey’s post hoc test or Welch’s ANOVA with Games-Howell’s multiple comparisons test. K denotes ×1000. **P* < 0.05, ***P* < 0.01, ****P* < 0.001, *****P* < 0.0001.

**Figure S7. CNF-induced gut microbiota remodeling constrains CDCA-to-LCA conversion.** (A) Bile salt hydrolase (BSH) activity in fecal suspensions from the ICP, ICP-CNC, and ICP-CNF groups. (B) Fecal lithocholic acid (LCA)/chenodeoxycholic acid (CDCA) ratio and (C) deoxycholic acid (DCA)/cholic acid (CA) ratio *in vivo*. (D) *In vitro* conversion of CDCA to LCA by fecal microbiota. Data are presented as mean ± SEM (n = 5). Statistical analysis in (A) and (D) was performed using one-way ANOVA followed by Tukey’s multiple-comparisons test; data in (B) and (C) were analyzed using Welch’s ANOVA followed by Dunnett’s multiple-comparisons test. *P < 0.05, **P < 0.01, ***P < 0.001.

**Figure S8. Nanocellulose-mediated alterations in key metabolites along the gut-liver axis.** (**A**) PLS-DA models evaluating the discriminatory power of metabolites in fecal, gut, serum, and liver samples. (**B**) Venn diagrams and Upset plots illustrating the distribution of differentially abundant metabolites in the NP, UDCA, and nanocellulose groups versus the ICP group for all sample types. Dots represent shared or unique metabolite intersections across comparisons. (**C**) An alternative Venn diagram depicting the overlap of differentially abundant metabolites from the ICP–CNF vs. ICP and NP vs. ICP comparisons. (**D**) Heatmap showing group-specific changes in tryptophan-, indole-, and TCA cycle–related metabolites detected in fecal, gut, serum, and liver samples. Red blocks indicate higher levels, and blue blocks indicate lower levels in the numerator group relative to the denominator group. Only metabolites with P < 0.05 are shown.

**Figure S9. Metabolic signatures and metabolic pathway analysis associated with nanocellulose intervention.** (**A**) Heatmaps of amino acid- and fatty acid-related metabolites detected in feces, gut, serum, and liver, showing group-wise differences. Red and blue indicate increased and decreased levels, respectively, in the numerator group compared to the denominator group. Only metabolites with P < 0.05 are shown. (**B**) Serum levels of selected representative metabolites. Data are presented as mean±SEM. *P* values were determined by one-way ANOVA with Tukey’s correction. **P* < 0.05, ***P* < 0.01, ****P* < 0.001, *****P* < 0.0001. (**C**) Predicted changes in metabolic pathway activities in fecal and serum samples between the ICP–CNF and ICP groups, shown as log₂ fold changes. Dashed lines indicate the baseline of the reference group (set to 0). Red dots above and below the baseline represent upregulated and downregulated activity, respectively. Dot size reflects the pathway enrichment ratio, and color intensity corresponds to the statistical significance. Only pathways with P < 0.05 (logistic regression) and q < 0.1 (FDR-adjusted) are included. (**D**) Sankey diagram highlighting key metabolites enriched in bile acid biosynthesis, tryptophan metabolism, and the TCA cycle in fecal and serum samples.

**Figure S10. Correlations between gut microbiota and ICP-related clinical and metabolic features.** (**A**) Spearman correlation between gut microbial taxa and clinical indicators relevant to ICP. b–d, Spearman correlation between differentially abundant metabolites in fecal (**B**), serum (**C**), and liver (**D**) samples and microbial composition. Red upward ellipses indicate positive correlations; blue downward ellipses indicate negative correlations. **P* < 0.05, ***P* < 0.01, ****P* < 0.001, *****P* < 0.0001.

**Figure S11. ASBT protein expression in the distal ileum.** Immunofluorescence staining of ASBT (red) in the distal ileum across five groups. Nuclei were counterstained with DAPI (blue). **P* < 0.05.

**Figure S12. *In vitro* validation of the intestinal FXR-FGF19-hepatic FXR signaling axis.** (A) mRNA expression of FXR signaling-related genes in Caco-2 cells treated with CDCA or GW4064. (B) ASBT protein expression in Caco-2 cells. (C) FGF19 levels in the culture supernatant of Caco-2 cells after CDCA or GW4064 treatment. (D) mRNA expression of hepatic FXR target genes in primary human hepatocytes exposed to conditioned media or GW4064. (E) CYP7A1 and MRP2 protein expression in hepatocytes. Data are presented as mean ± SEM. Statistical significance was determined by one-way ANOVA followed by Tukey’s multiple-comparisons test. *P < 0.05, **P < 0.01, ***P < 0.001, ****P < 0.0001.

**Figure S13.** Atomic force microscopy (AFM) images of the bacterial suspensions from the ICP-CNC (A) and ICP-CNF (B) donor groups used for FMT. No CNC or CNF residues were detected in the suspensions.

**Supplementary Tables**

**Table S1.** Analysis of TBA, AST, and ALT levels in the ICP rat model following nanocellulose intervention

| **Groups** | **TBA** | **AST** | **ALT** |
| --- | --- | --- | --- |
| N=6 | (umol/L) | (U/L) | (U/L) |
| **NP** | 12.6±1.61^***^ | 142.33±11.11^***^ | 82.6±21.3 |
| **ICP** | 86.82±15.04 | 182.38±11.72 | 76.1±29.79 |
| **ICP-UDCA** | 82.4±31.09 | 177.5±11.6 | 53.17±10.97 |
| **ICP-CNC** | 65.62±5.74 | 168.25±11.9 | 48.15±9.69 |
| **ICP-CNF** | 27.31±9.74^***^ | 143.38±17.83^***^ | 45.83±11.92 |

Data are presented as mean ± SEM. *P* values were determined by one-way ANOVA with Tukey’s correction or Welch’s ANOVA with Games-Howell’s test. ****P* < 0.001 versus ICP group.

**Table S2.** Bile acid standards

Glycocholic Acid (GCA)	#ZS-20137

Glycochenodeoxycholic Acid (GCDCA)	#ZC-53938

Taurocholic Acid (TCA)	#ZS-20134

Taurochenodeoxycholic Acid (TCDCA)	#ZS-20033

Glycoursodeoxycholic Acid (GUDCA)	#IR-15585

Ursodeoxycholic Acid (UDCA)	#ZS-20012

Deoxycholic Acid (DCA)	#ZC-53569

Tauroursodeoxycholic Acid (TUDCA)	#ZC-50900

Hyodeoxycholic Acid (HDCA)	#ZS-20019

Glycodeoxycholic Acid (GDCA)	#IR-15374

Glycolithocholic Acid (GLCA)	#ZS-20046

Alpha-muricholic Acid (α-MCA)	#ZS-20001

Beta-muricholic Acid (β-MCA)	#ZS-20003

Omega-murichoclic Acid (ω-MCA)	#ZS-20006

Murideoxycholic Acid (MDCA)	#ZS-20309

Taurohyocholic Acid (THCA)	#ZS-20025

Taurolithocholic Acid (TLCA)	#ZS-20049

Taurodeoxycholate Acid (TDCA)	#ZS-20147

Lithocholic Acid (LCA)	#ZS-20043

Cholic Acid (CA)	#ZC-53439

Chenodeoxycholic Acid (CDCA)	#ZT-71935

Hyocholic Acid (HCA)	#ZC-26210

Glycodehydrocholic Acid (GDHCA)	#ZS-20161

Glycohyocholic Acid (GHCA)	#ZS-21537

Tauro-alpha-muricholic Acid (T-α-MCA)	#ZS-20002

Tauro-beta-muricholic Acid (T-β-MCA)	#ZS-20005

Glycohyodeoxycholic Acid (GHDCA)	#ZC-57516

**Table S3.** Standard rat maintenance diet formulation

| SPF-grade rat maintenance diet | | | |
| --- | --- | --- | --- |
| Moisture ≤ 100g | Iron ≥ 100mg | Choline ≥ 1250mg | Valine ≥ 8.4g |
| Crude Protein ≥ 180g | Manganese ≥ 75mg | Lysine ≥ 8.2g | Vitamin A ≥ 7000 IU |
| Crude Fat ≥ 40g | Copper ≥ 10mg | Egg + Cysteine ≥ 5.3g | Vitamin D ≥ 800 IU |
| Crude Fiber ≤ 50g | Zinc ≥ 30mg | Arginine ≥ 9.9g | Vitamin E ≥ 60 IU |
| Ash ≤ 80g | Iodine ≥ 0.5mg | Histidine ≥ 4.0g | Vitamin K ≥ 3.0mg |
| Calcium 10-18g | Selenium ≥ 0.1-0.2mg | Tryptophan ≥ 1.9g | Vitamin B1 ≥ 8mg |
| Total Phosphorus 6-12g | Niacin ≥ 45mg | Phenylalanine + Tyrosine ≥ 11.0g | Vitamin B2 ≥ 10mg |
| Magnesium ≥ 2.0g | Pantothenic acid ≥ 17mg | Threonine ≥ 6.5g | Vitamin B6 ≥ 6mg |
| Potassium ≥ 5.0g | Folic acid ≥ 4.00mg | Leucine ≥ 14.4g | Vitamin B12 ≥ 0.020mg |
| Sodium ≥ 2.0g | Biotin ≥ 0.10mg | Isoleucine ≥ 7.0g |  |

**Table S4.** Primer sequences for real-time quantitative PCR of rat genes

| Target  genes | Forward sequences (5‘-3‘) | Reverse sequences (5‘-3‘) |
| --- | --- | --- |
| *Cyp7a1* | CAGGTCTCTGAACTGATCCGTCTAC | AGAATAGCGAGGTGCGTCTTGG |
| *Cyp27a1* | GGAGCACCGAGACCACAAAGG | CCTCAGCAGGCTTCAGCATCC |
| *Cyp8b1* | CCCATCATTAAGAGCACGCAGAAAG | CAGCATCTGGTGGTCCTCATCC |
| *Ntcp* | TACTGGCTACCTCCTCCCTGATG | TGGATTGAGTTGGAAGAGAGCAGAG |
| *Bsep* | AGTGGTGGTCAGAAGCAAAGAGTAG | TGAGGTAGCCATATCCAGAAGCAAG |
| *Mrp2* | TCTCGGTCTTATGCGGCGTATTC | GACGAAGAACAGGTAGGAGTAGGC |
| *Asbt* | GCATTTCTCTGGTTGCGCTT | TCGCTTTTTGGGGCCATTTG |
| *Ibabp* | GAGGTCGTGGGTGACAAGTT | AGCCACCCTCTTGCTTACAC |
| *Fxr* | TGGACTCTATGAACTCAGGCGAATG | TTGTCACAGGCATCTCGGATACC |
| *Shp* | CAGGAGGCTCACTGGGCATTG | CGATGACAGGGCGGAAGAAGAG |
| *Fgf15* | ACGGCAAGATATACGGGCTG | GATGTGGAGGTGGTGCTTCA |
| *β-actin* | TGTCACCAACTGGGACGATA | GGGGTGTTGAAGGTCTCAAA |

**Table S5.** Primer sequences for real-time quantitative PCR of human genes

| Target  genes | Forward sequences (5‘-3‘) | Reverse sequences (5‘-3‘) |
| --- | --- | --- |
| *FXR* | GGGGCAACTGTGTGATGGAT | CCAACATTCCCATCTCTTTGCAT |
| *SHP* | TCTTCAACCCCGATGTGCC | ACCAGGGTTCCAGGACTTCA |
| *FGF19* | AGATCAAGGCAGTCGCTCTG | GAGTACTGAAGCAGCCCCTG |
| *CYP7A1* | GCCTGCGCATGTTTCTCAAT | TGGAATGGTGTTTGCTTGCG |
| *MRP2* | GAGGCAGACCTGCCACTTT | AGGAGCCATAGGTAGCCCAA |
| *ASBT* | GTTACTCCCTGGGGTTTCTTCTGG | GAGCGGGAAGGTGAATACGACA |
| *GAPDH* | TCTCCTCTGACTTCAACAGCGAC | CCCTGTTGCTGTAGCCAAATTC |
